# Oestrogen enforces the integrity of blood vessels in the bone during pregnancy and menopause

**DOI:** 10.1038/s44161-022-00139-0

**Published:** 2022-10-12

**Authors:** Julia Rodrigues, Yi-Fang Wang, Amit Singh, Michelle Hendriks, Gopuraja Dharmalingam, Martine Cohen-Solal, Anjali Kusumbe, Saravana K. Ramasamy

**Affiliations:** 1 Institute of Clinical Sciences, Imperial College London, London W12 0NN, UK; 2 MRC London Institute of Medical Sciences, Imperial College London, London W12 0NN, UK; 3 Bioinformatics and computing Facility, MRC London Institute of Medical Sciences, Imperial College London, UK; 4 Tissue and Tumor Microenvironments Group, MRC Human Immunology Unit, MRC Weatherall Institute of Molecular Medicine, University of Oxford, Oxford, UK; 5 Heidelberg University Biochemistry Center, University of Heidelberg, Heidelberg D-69120, Germany; 6 Bioscar Inserm U1132 and Université de Paris, Hospital Lariboisiere, Paris, France

**Keywords:** Ageing, oestrogen, postmenopausal bone loss, blood vessels, sex differences

## Abstract

The mammalian skeletal system shows sex differences in structure, functions, ageing and disease incidences. The role of blood vessels in physiological, regenerative and pathological bone functions indicates the requisite to understanding their sex specificity. Here, we find oestrogen regulates blood vessel physiology during pregnancy and menopause through oestrogen receptor alpha (ERα) and G-protein coupled oestrogen receptor-1 (Gper1) but not ERβ-dependent signalling in mice. Oestrogen regulates BECs’ lipid use and promotes lipolysis of adipocytes and FA uptake from the microenvironment. Low oestrogen conditions skew endothelial FA metabolism to accumulate lipid peroxides (LPO), leading to vascular ageing. High ferrous ion levels in female BECs intensify LPO accumulation and accelerate the ageing process. Importantly, inhibiting LPO generation using liproxstatin-1 in aged mice significantly improved bone heath. Thus, our findings illustrate oestrogen’s effects on BECs and suggest LPO targeting could be an efficient strategy to manage blood and bone health in females.

Changes in the skeletal system during puberty, pregnancy and menopause are controlled by sex hormones which directly or indirectly regulate physiological and pathological bone remodelling^1, 2^. The role of sex hormones on mesenchymal cells and haematopoietic cells has been well recognised^3, 4^. Despite playing a central role in skeletal physiology by regulating development, repair, ageing and disease^5–7^, the extent to which blood vessel functions differ between sexes remains poorly understood. In this study, we understand blood vessel changes during pregnancy and menopause and investigate the role of steroid hormones in mediating bone endothelial cells’ (BECs’) growth and ageing.

## Vascular changes in bone during pregnancy and menopause

**Extended Data Fig. 1:**
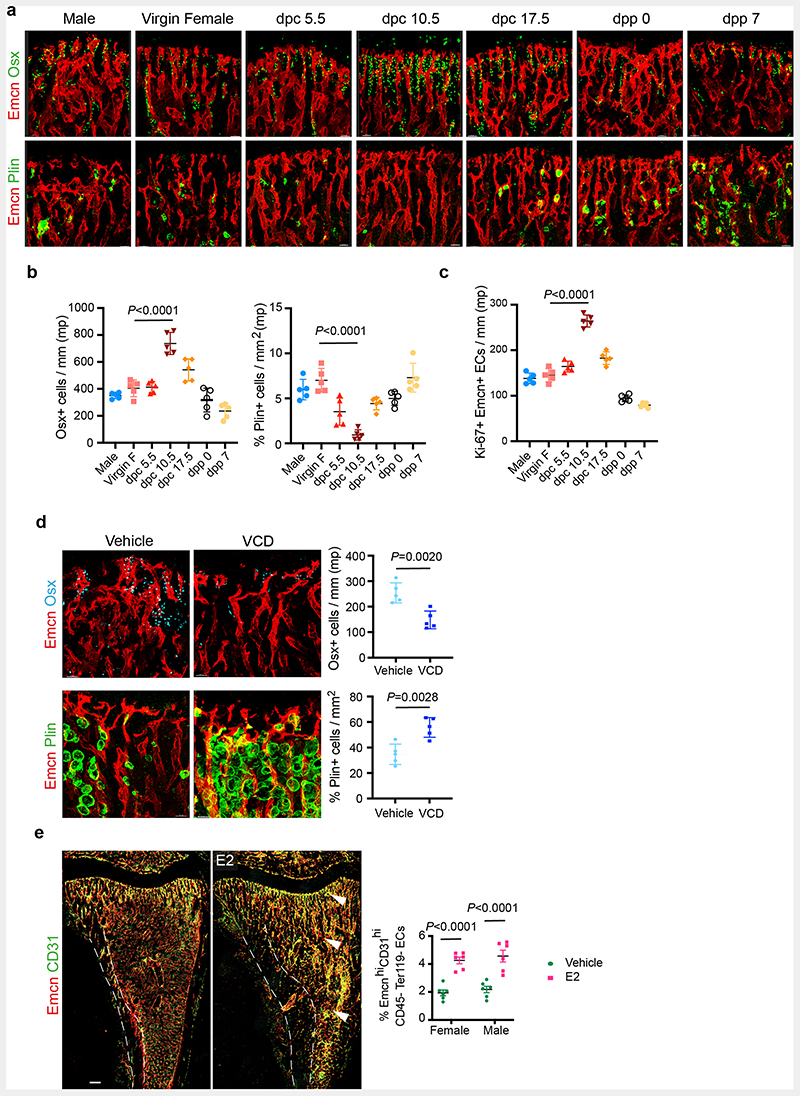
Changes in the frequency of OBLs and adipocytes during pregnancy and menopause **a**, Representative confocal images showing changes in OBLs (Osterix+) and bone marrow (BM) adipocytes (Perilipin+) at different time-points during mouse pregnancy and *post-partum*. Scale bars 40 μm. **b**, Quantification of OBLs and BM adipocytes described in **a**, normalised to metaphysis (mp) length and area respectively (n=5). Data are mean ± s.d.; One-Way ANOVA with Tukey’s test **c**, Quantification of Ki-67+ ECs at different time-points during mouse pregnancy, normalised to the length of mp (n=5). Data are mean ± s.d.; One-Way ANOVA with Tukey’s test **d**, Representative confocal images with quantifications (n=5) showing loss of OBLs, and increase in BM adipocytes in the VCD model. Data are mean ± s.d.; Unpaired two-tailed t-test. Scale bars 40 μm. **e**, Representative confocal tile scans of Vehicle and E2-treated bones at P28, illustrating increased Type-H vessels (arrowheads) in E2-treated mice. White dotted lines separate bone marrow from the cortical bone region. Quantification of Type-H ECs (CD31^high^ Emcn^high^) characterised by flow cytometry (n=6). Data are mean ± s.e.m.; Two-way ANOVA with Tukey’s test. Scale bars 100 μm.

**Fig. 1:**
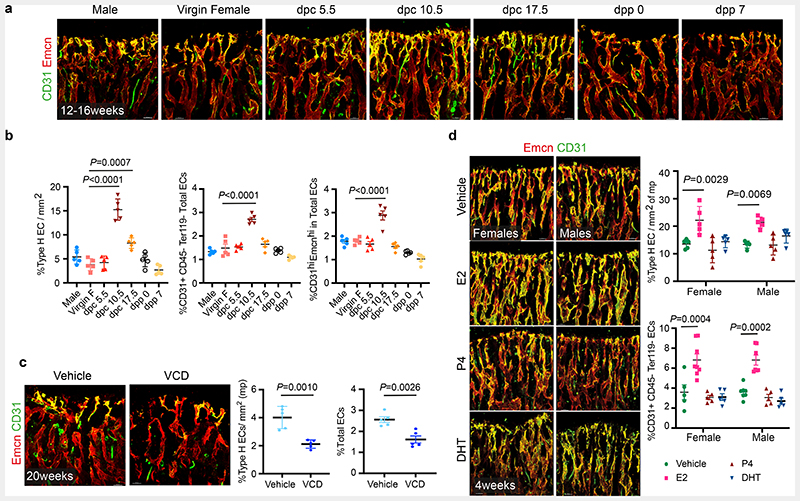
Oestrogen regulates blood vessel growth in bones **a**, Representative confocal images of Type-H (Emcn^high^ CD31^high^) vessel changes in maternal bones at different time-points of mouse pregnancy and post-delivery. Pregnancy time points are represented in *days post-coitum* (dpc) and post-delivery days as *days post-partum* (dpp). Scale bars 40μm **b**, Quantification of Type-H vessels described in **a**, as area of Type-H ECs normalised to area of metaphysis (mp) (n=5); total ECs (CD31+ CD45-Ter119-) and Type-H ECs (CD31^high^ Emcn^high^) characterised by flow cytometry (n=5). Data are mean ± s.d. for image quantification and mean ± s.e.m. for flow cytometry data; One-Way ANOVA with Tukey’s test **c**, Representative confocal images of vehicle and VCD (n=5) model mice (20 weeks old) depicting reduced Type-H vessels in VCD model. Scale bars 40μm. Graphs indicate the area of Type-H ECs normalised to the area of mp and total ECs quantified by flow cytometry. Data are mean ± s.d. for image quantification and mean ± s.e.m. for flow cytometry data; Unpaired two-tailed t-test **d**, Representative confocal images showing changes in Type-H vessels in male and female mice (P28) upon hormonal treatments with quantification for Type-H ECs, area quantification (n=5); flow cytometry (Vehicle n=5F, n=7M; E2 n=8F, n=7M; P4 n=5F, n=5M; DHT n=5F, n=5M). Data are mean ± s.d. for image quantification and mean ± s.e.m. for flow cytometry data; Two-Way ANOVA with Tukey’s test. Scale bars 40μm.

To understand cellular changes in the maternal bone microenvironment (BM) during pregnancy, we analysed the tibia of pregnant mice on different *days post-coitum* (dpc) using improved imaging methods ^8^. We used endomucin and CD31 to label blood vessels, osterix to mark early osteoblast lineage cells (OBL) and perilipin to identify adipocytes in the BM. CD31^high^/ Endomucin^high^ expressing Type-H capillaries are angiogenic blood vessels that support osteogenesis in the bone ^9, 10^. We observed a notable increase in type-H vessels and OBLs, and a decline in adipocytes in bones of 10.5 dpc pregnant dams compared to littermate virgins, males and other pregnant stages (Fig. 1a, Extended Data Fig. 1a and b). Increased percentage of total CD31+CD45-Ter119-endothelial cells (ECs), CD31^high^/ Endomucin^high^ (type-H) ECs within total ECs and proliferating (Ki67+) ECs confirmed the promotion of angiogenesis on 10.5 dpc (Fig. 1b, Extended Data Fig. 1c and Supplementary Figure 1).

To understand cellular changes in BMs during menopause, we generated a menopause mouse model by injecting Vinylcyclohexene diepoxide (VCD) in 12-weeks-old females. Injection of VCD for 20 days and analysis after 32 days confirmed complete depletion of ovarian follicles (Supplementary Figure 2), which modelled human menopause conditions ^11, 12^. Bones of menopause mice showed diminished total and type-H BECs (Fig. 1c) in addition to reduced OBLs and increased adipocytes (Extended Data Fig. 1d). These findings show that pregnancy and menopause triggered significant changes in endothelial and mesenchymal compartments within the BM.

## Oestrogen regulates the growth of blood vessels in the bone

**Extended Data Fig. 2:**
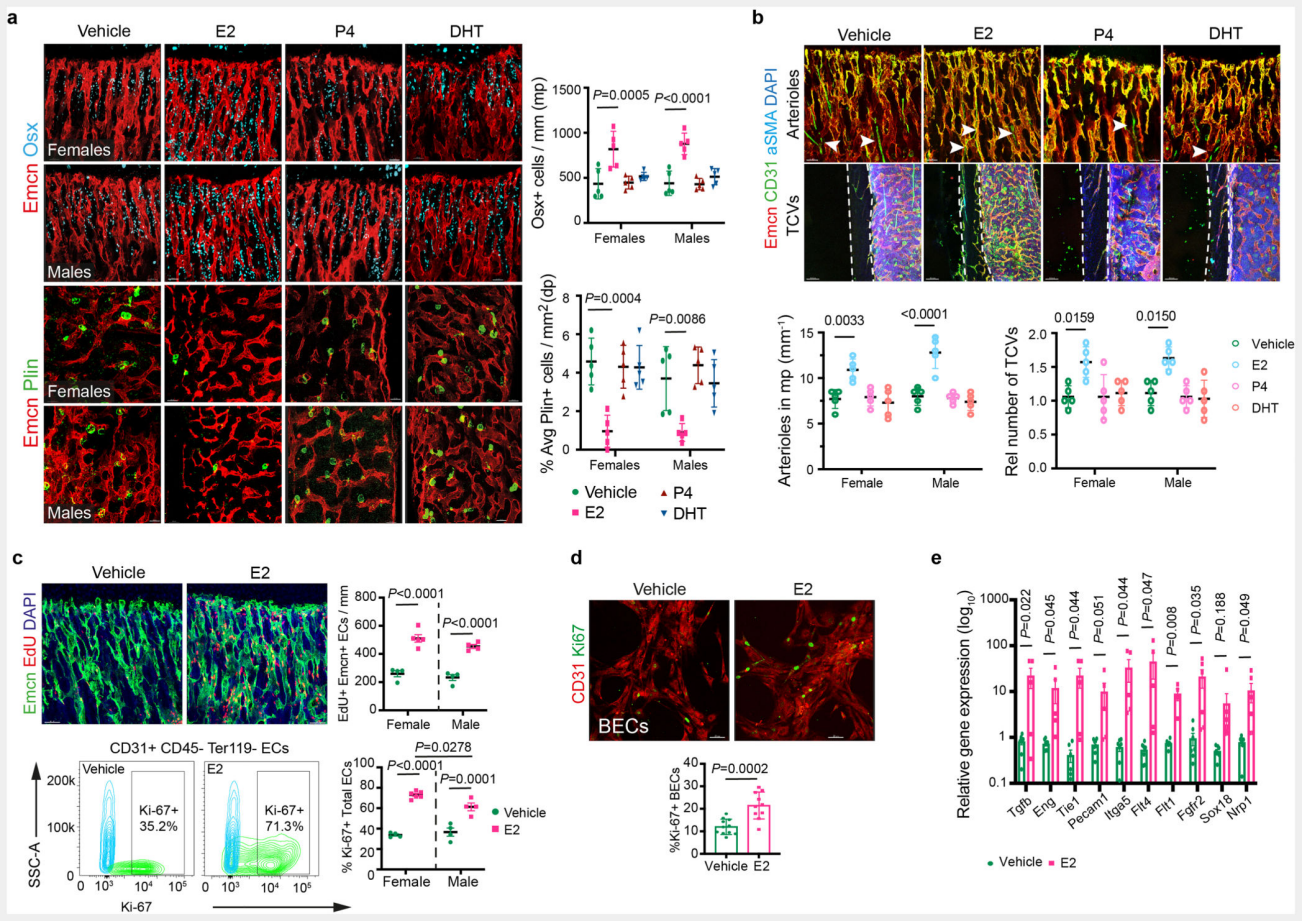
Oestrogen-mediated changes in bone endothelium **a**, Representative confocal images with quantifications of OBLs (Osterix+, n=5) and BM adipocytes (Perilipin+, n=5) of mice treated with different hormones at P28. Data are mean ± s.d.; Two-way ANOVA with Tukey’s test. Scale bars 40 μm. **b**, Representative confocal images show endomucin (Emcn, red), CD31 (green), alpha-smooth muscle actin (αSMA, cyan) blood vessels in the metaphysis and cortical bone area. Graphs show quantification of CD31+ Emcn-arterioles (white arrowheads) and TCVs in mice treated with Vehicle, E2, P4 and DHT (n=5); Data are mean ± s.d.; Two-way ANOVA with Tukey’s test. White dotted lines denote the cortical bone region. DAPI is a nuclear counterstain. Scale bars 100 μm (TCVs); 50 μm (arterioles). **c**, Representative confocal images of EdU-injected Vehicle and E2-treated mice at P28, with quantification of EdU+ ECs normalised to metaphysis (mp) length (Vehicle n=4F, n=4M; E2 n=5F, n=4M) indicating increased EdU+ ECs with E2 treatment. Scale bars 50 μm. Representative flow cytometry plots of Ki-67+ ECs, with quantification of Ki-67+ ECs in bone (Vehicle n=4F, n=4M; E2 n=6F, n=4M) indicating an increase in Ki-67+ ECs with E2 treatment. Image quantification data are mean ± s.d. with Two-way ANOVA with Tukey’s test, and flow cytometry data mean ± s.e.m. with Two-way ANOVA with Tukey’s test. **d**, Representative confocal images and quantification of Ki-67+ nuclei of Vehicle and E2-treated BECs (Vehicle n=11, E2 n=10) with quantification of average from multiple 20x fields of view indicating an increase in Ki-67+ cells with E2 treatment. Data are mean ± s.d.; Unpaired two-tailed t-test. Scale bars 50 μm **e**, Graph showing relative gene expression for angiogenic genes in Vehicle and E2-treated mice, indicating an increase in angiogenesis upon E2 treatment. (*Tgfb* Vehicle n=8, E2 n=5; *Eng* Vehicle n=8, E2 n=5; *Tie1* Vehicle n=6, E2 n=5; *Pecam1* Vehicle n=8, E2 n=5; *Itga5* Vehicle n=7, E2 n=5; *Flt4* Vehicle n=8, E2 n=5; *Flt1* Vehicle n=5, E2 n=4; *Fgfr2* Vehicle n=7, E2 n=6; *Sox18* Vehicle n=8, E2 n=8; *Nrp1* Vehicle n=7, E2 n=6.) Data are mean ± s.e.m.; Multiple Unpaired two-tailed t-test

**Extended Data Fig. 3:**
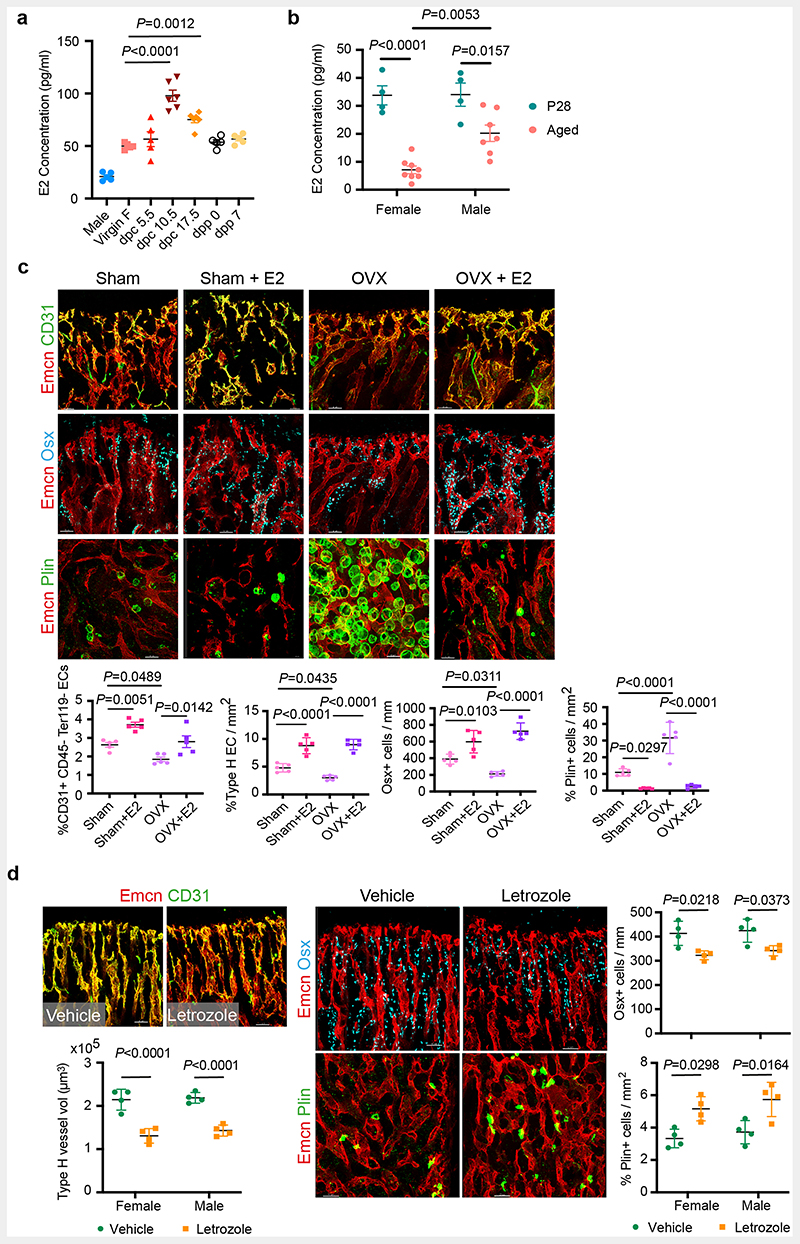
Oestrogen-mediated changes in bone endothelium **a**, Graph showing serum E2 concentration at different time-points during mouse pregnancy (n=6 for dpc10.5, dpc17.5; n=5 for Male, Virgin F, dpc5.5, dpp0, dpp7). Data are mean ± s.e.m.; One-Way ANOVA with Tukey’s test **b**, Graph showing serum E2 concentration in P28 (n=4F, n=4M) and aged (n=8F, n=7M) mice. Data are mean ± s.e.m.; Two-Way ANOVA with Tukey’s test **c,** Representative confocal images with quantifications of Type-H (yellow, Emcn^high^ CD31^high^) blood vessels (n=5 for all conditions), OBLs (Osterix+, n=5), and BM adipocytes (Perilipin+, n=5) in Vehicle and E2-treated sham-operated and OVX mice. Flow cytometry quantification of total ECs (CD31+CD45-Ter119-) shows the recovery of BECs in E2-treated bones (n=5). Data are mean ± s.d. for image quantification and mean ± s.e.m. for flow cytometry data. Two-Way ANOVA with Tukey’s test. Scale bars 50μm **d**, Bone tissue sections of Vehicle and Letrozole-treated mice at P28 were immunostained with CD31 (green) and Emcn (red) to identify type-H vessels. The graph shows quantifications of Type-H (yellow) blood vessels (n=4). The distribution of OBLs and BM adipocytes in these bone sections were imaged using Osterix+ (cyan) and Perilipin (green). Graphs show quantification of OBLs (n=4) and adipocytes (n=4) in Vehicle and Letrozole-treated mice. Data are mean ± s.d.; Two-Way ANOVA with Tukey’s test. Scale bars 50μm

To understand the contribution of sex hormones in these cellular changes, we administered sex hormones, oestradiol (E2; 17β-oestradiol, 2ug/day), progesterone (P4; 1ug/day) and dihydrotestosterone (DHT; 100ug/day) to young male and female mice for 10 days and analysed their BMs after 2 weeks. Systemic treatment with E2 resulted in the accumulation of type-H capillaries in the bone marrow compartment. We observed a higher frequency of total and type-H ECs in bones of both sexes compared to P4- and DHT-administered bones (Fig. 1d and Extended Data Fig. 1e). Increase in OBLs and decline in adipocytes of E2 treated bones indicated that cellular changes were similar to pregnancy-associated changes. While OBLs showed a marginal increase in DHT treatment, adipocytes were unaltered in both P4 and DHT treatments (Extended Data Fig. 2a). In addition to type-H capillaries, E2 promoted arteriolar (CD31+ Emcn-vessels) and transcortical vessels (TCVs) in E2-treated bones. Capillary subtypes were unaltered in P4 and DHT treatments (Extended Data Fig. 2b). Analysis of EC proliferation using 5-Ethynyl-2'-deoxyuridine (EdU) labelling and Ki67 immunostaining showed increased incorporation of EdU and frequency of Ki67+ ECs in E2 treated bones. Likewise, cultured primary BECs showed increased proliferation *in vitro* upon E2 treatment (10μM) (Extended Data Fig. 2c,d). We also analysed transcript levels of angiogenic factors in purified BECs to understand angiogenesis. Higher expression of known angiogenic regulators in E2 treated BECs compared to vehicle controls supported the promotion of angiogenesis in bones by elevated E2 levels (Extended Data Fig. 2e). Quantification of E2 levels at different stages of pregnancy (Extended Data Fig. 3a) confirmed elevated endogenous oestrogen levels on 10.5dpc pregnant dams corresponding with the status of angiogenesis in their bones. Similarly, ageing reduced circulating E2 levels in mice (Extended Data Fig. 3b). To study blood vessel changes in bones under systemic low E2 conditions, we used ovariectomised mouse models. Ovariectomy has been an established method to reduce circulating E2 levels and promote bone loss^13^. Ovariectomy was performed in 8-weeks-old female mice, and bones were analysed after 6 weeks. Ovariectomized (OVX) mice demonstrated vascular and mesenchymal phenotypes similar to ovarian follicle depletion. Recovery of bone phenotypes in OVX mice upon E2 administration established the central role of oestrogen in this phenotype (Extended Data Fig. 3c). We also used pharmacological aromatase inhibitor, Letrozole to arrest oestrogen synthesis and thus reduce circulating E2 levels. Administration of Letrozole (100ug/day) in mice reduced angiogenic type-H capillaries and OBLs, and increased adipocytes in developing bones (Extended Data Fig. 3d). These findings established the role of oestrogen in regulating the growth of blood vessels in the bone.

## Oestrogen Receptor signalling in BECs

**Extended Data Fig. 4:**
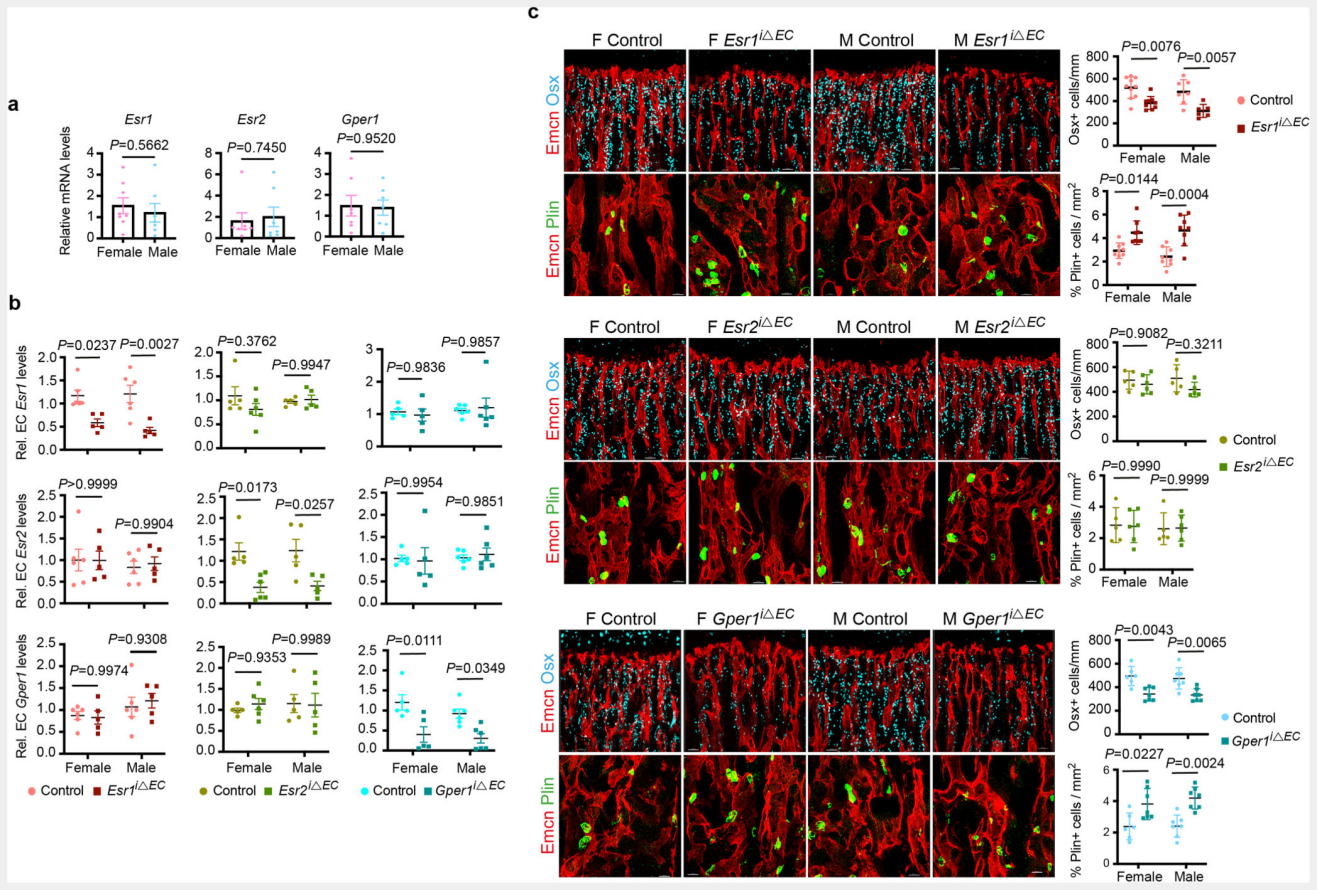
Cellular changes in the bone microenvironment of endothelial-specific oestrogen receptor deletion mutants **a**, Graphs indicating relative mRNA levels of oestrogen receptors (*Esr1*, *Esr2*, *Gper1*) in female and male BECs at P28. (*Esr1*: n=8F, n=7M; *Esr2*, *Gper1*: n=7) Data are mean ± s.e.m.; Unpaired two-tailed t-test **b**, Graphs showing ER transcript levels in purified BECs of endothelial *Esr1*, *Esr2*, or *Gper1* deletion mutant and control littermates (*Esr1* Control n=6F, n=6M, Mutant n=5F, n=5M; *Esr2* Control n=5F, n=5M, Mutant n=6F, n=5M; *Gper1* Control n=5F, n=6M, Mutant n=5F, n=6M). Data are mean ± s.e.m.; Two-Way ANOVA with Tukey’s test **c**, Representative confocal images of mutants with endothelial *Esr1*, *Esr2*, or *Gper1* deletion compared to littermate Cre-controls, with quantifications showing loss of OBLs, and increase in adipocytes after *Esr1* and *Gper1* deletion (*Esr1* :Osx-Control n=10F, n=7M, Mutant n=9F, n=6M; *Esr1* :Plin-Control n=9F, n=8M, Mutant n=8F, n=8M; *Esr2*:Osx-Control n=5F, n=5M, Mutant n=6F, n=5M; *Esr2*:Plin Control n=5F, n=5M, Mutant n=6F, n=6M; *Gper1*: both Osx and Plin-Control n=6F, n=7M, Mutant n=7F, n=7M). Data are mean ± s.d.; Two-Way ANOVA with Tukey’s test. Scale bars 40μm

**Extended Data Fig. 5:**
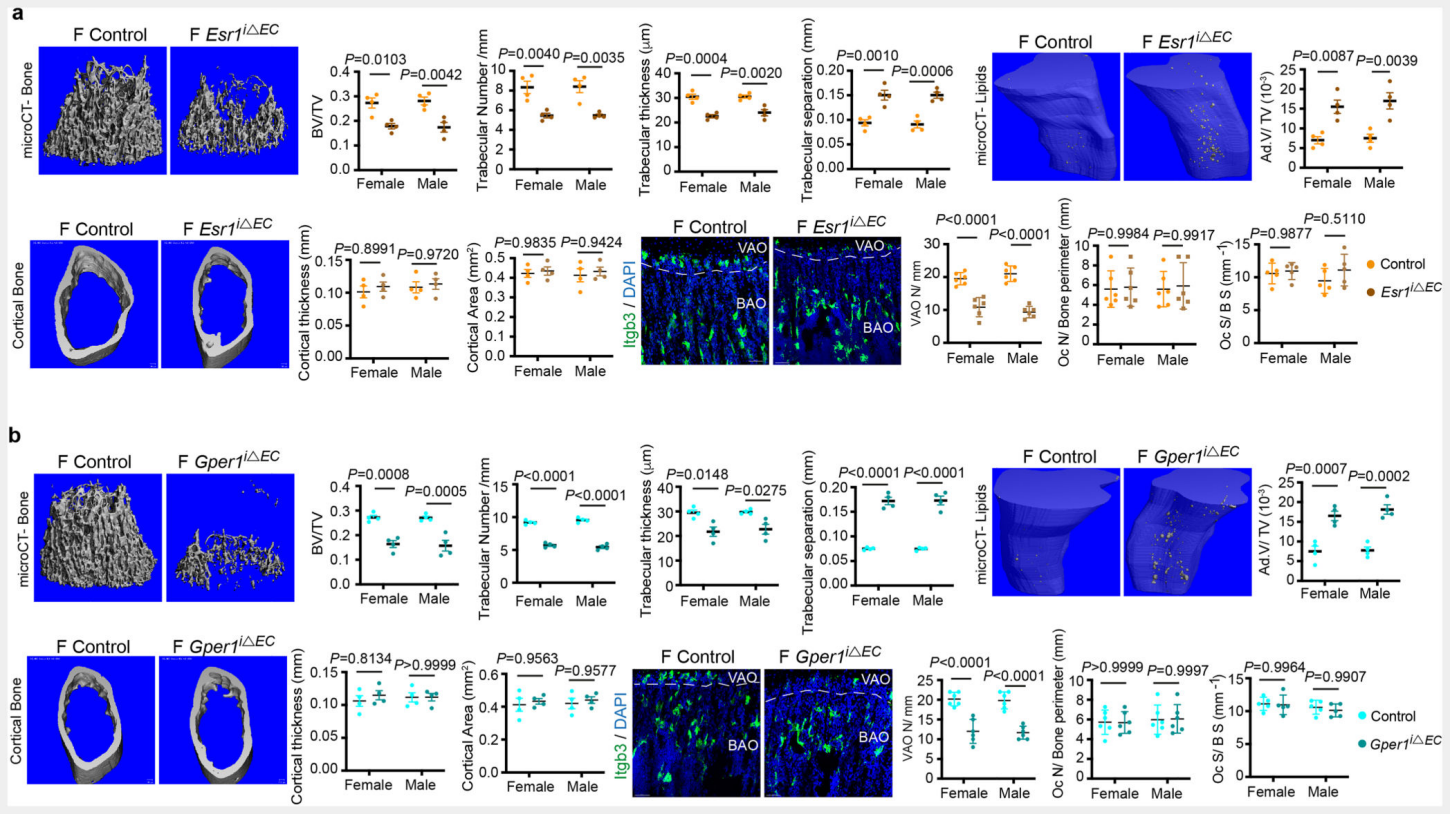
Phenotype analysis of endothelial ER mutants **a**, Representative microCT analysis of mutants with endothelial *Esr1* deletion compared to littermate Cre-controls, with quantifications showing trabecular bone volume fraction (BV/TV), number, thickness and separation (n=4), quantification of adipocytes (n=4) and cortical bone quantification of thickness and area (n=4). Representative confocal images show osteoclasts distribution in control and mutant bones with quantification of osteoclasts number (n=6) and coverage (n=5). Data are mean ± s.e.m. for micro-CT results and ± s.d for image quantification; Two-Way ANOVA with Tukey’s test. Scale bars 100μm (microCT), 50μm (confocal) **b**, Representative microCT images of mutants with endothelial *Gper1* deletion compared to littermate Cre-controls, with quantifications showing trabecular bone volume fraction (BV/TV), number, thickness, and separation (n=4), quantification of adipocytes (n=4) and cortical bone quantification of thickness and area (n=4). Representative confocal images show osteoclasts distribution in control and mutant bones with quantification of osteoclasts number (n=6) and coverage (n=5). Data are mean ± s.e.m. for micro-CT results and ± s.d for image quantification; Two-Way ANOVA with Tukey’s test. Scale bars 100μm (microCT), 50μm (confocal)

**Fig. 2:**
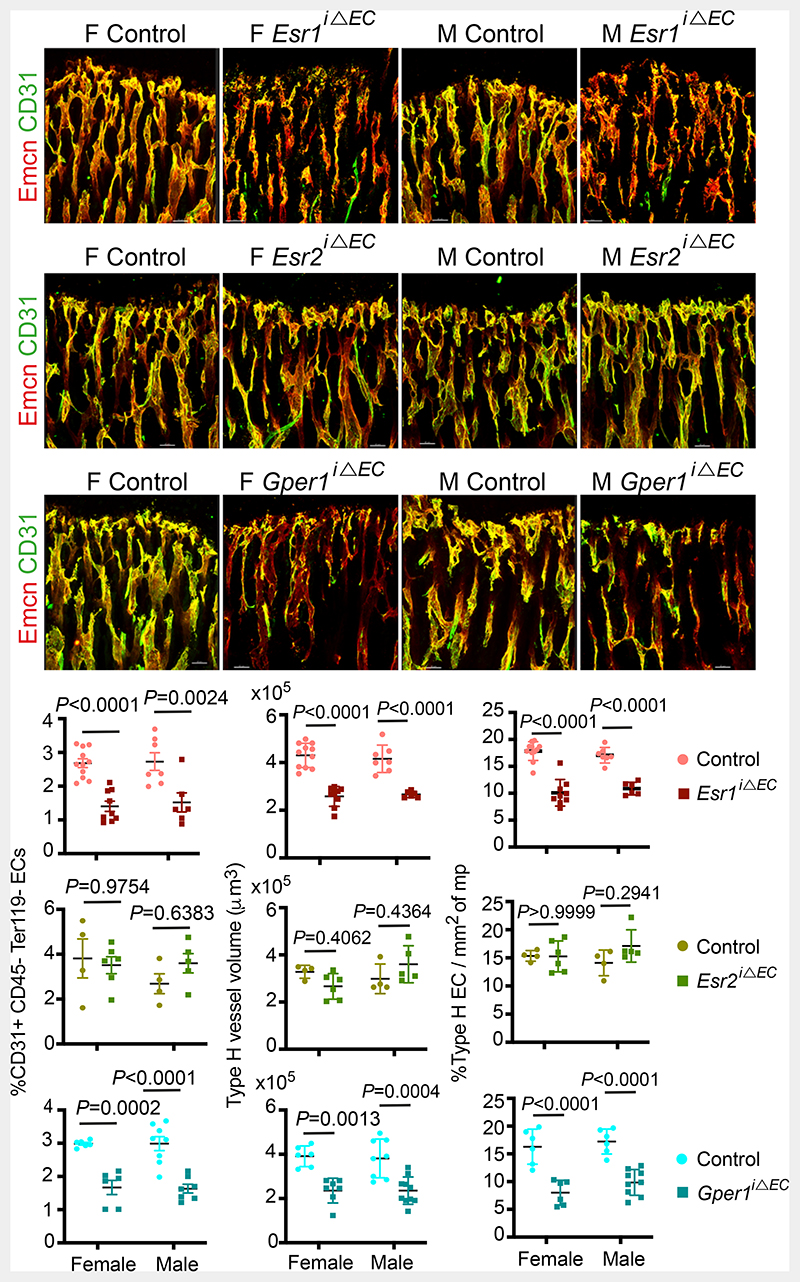
Oestrogen signalling is mediated via oestrogen receptor 1 (*Esr1*) and G-protein coupled oestrogen receptor 1 (*Gper1*) Representative confocal images of Type-H (Emcn^high^ CD31^high^) vessel changes upon endothelial-specific deletion of oestrogen receptors (*Esr1*, *Esr2*, *Gper1*) at P28 in mice. Graphs indicate quantifications of total ECs (CD31+ CD45-Ter119-) by flow cytometry for *Esr1* (Control n=11F, n=7M; Mutant n=9F, n=6M), *Esr2* (Control n=4F, n=4M; Mutant n=6F, n=5M), *Gper1* (Control n=6F, n=8M; Mutant n=6F, n=7M); quantification of Type-H vessel volume per field of mp for *Esr1* (Control n=11F, n=7M; Mutant n=9F, n=6M), *Esr2* (Control n=4F, n=4M; Mutant n=6F, n=5M), *Gper1* (Control n=6F, n=8M; Mutant n=7F, n=10M); and quantification of Type-H EC area normalised to mp area for *Esr1* (Control n=11F, n=7M; Mutant n=9F, n=6M), *Esr2* (Control n=4F, n=4M; Mutant n=6F, n=5M), *Gper1* (Control n=6F, n=6M; Mutant n=6F, n=9M). Data are mean ± s.d. for image quantification and mean ± s.e.m. for flow cytometry data; Two-Way ANOVA with Tukey’s test. Scale bars 40μm.

To study endothelial-specific changes mediated by oestrogen, we investigated oestrogen receptor (ER) signalling in BECs. Transcript analysis for ERs in freshly isolated BECs showed expression of *Esr1* (ERα), *Esr2* (ERβ) and *Gper1* (G-protein coupled oestrogen receptor-1; Gper1) in both the sexes (Extended Data Fig. 4a). We investigated the functions of ERs in blood vessel growth by targeting individual receptors in BECs. We generated inducible EC-specific loss-of-function mice using loxP flanked either *Esr1* (*Esr1*^lox/lox^) ^14^ or *Esr2* (*Esr2*^lox/lox^) ^15^ or *Gper1(Gper1*^lox/lox^) ^16^ alleles with *Cdh5(PAC)-CreERT2* mouse line ^17^. We analysed postnatal (4-weeks-old) male and female bones to understand blood vessel changes. Gene deletions were confirmed by quantification of receptor transcript levels in purified BECs from control and mutant mice (Extended Data Fig. 4b). Deletion of one receptor showed minimal influence on the other receptors' transcript levels in BECs (Extended Data Fig. 4b). EC-specific loss of functions of *Esr1* and *Gper1* resulted in the reduction of total and type-H ECs, while *Esr2* mutants did not manifest endothelial phenotype in both the sexes (Fig. 2). Thus inhibition of oestrogen receptors, *Esr1* and *Gper1* impaired blood vessel growth in bones. We also observed a reduction of OBL numbers and an increase of adipocytes in *Esr1* and *Gper1* mutants (Extended Data Fig. 4c). We performed micro-computed tomography (micro-CT) analysis of *Esr1* and *Gper1* mutant bones to understand the changes at the whole organ level. Micro-CT data confirmed the reduction in trabecular bone and increase in lipid contents of both the endothelial mutants (Extended Data Fig. 5a and b). Further analysis showed unaltered cortical bone thickness and area in these endothelial mutants. Similarly, osteoclasts on bone surfaces were not altered by the deletion of ERs on ECs. Vessel-associated osteoclasts, a subtype of osteoclasts present at the osteochondral interface are dependent on angiogenic blood vessels^18^. The reduction of vessel-associated osteoclasts^18^ in the metaphysis supported the defective blood vessel growth in these mutants (Extended Data Fig. 5a and b). These findings demonstrated the functional role of ER signalling in BECs and changes in the BM.

## Metabolic regulation of BECs

**Extended Data Fig. 6:**
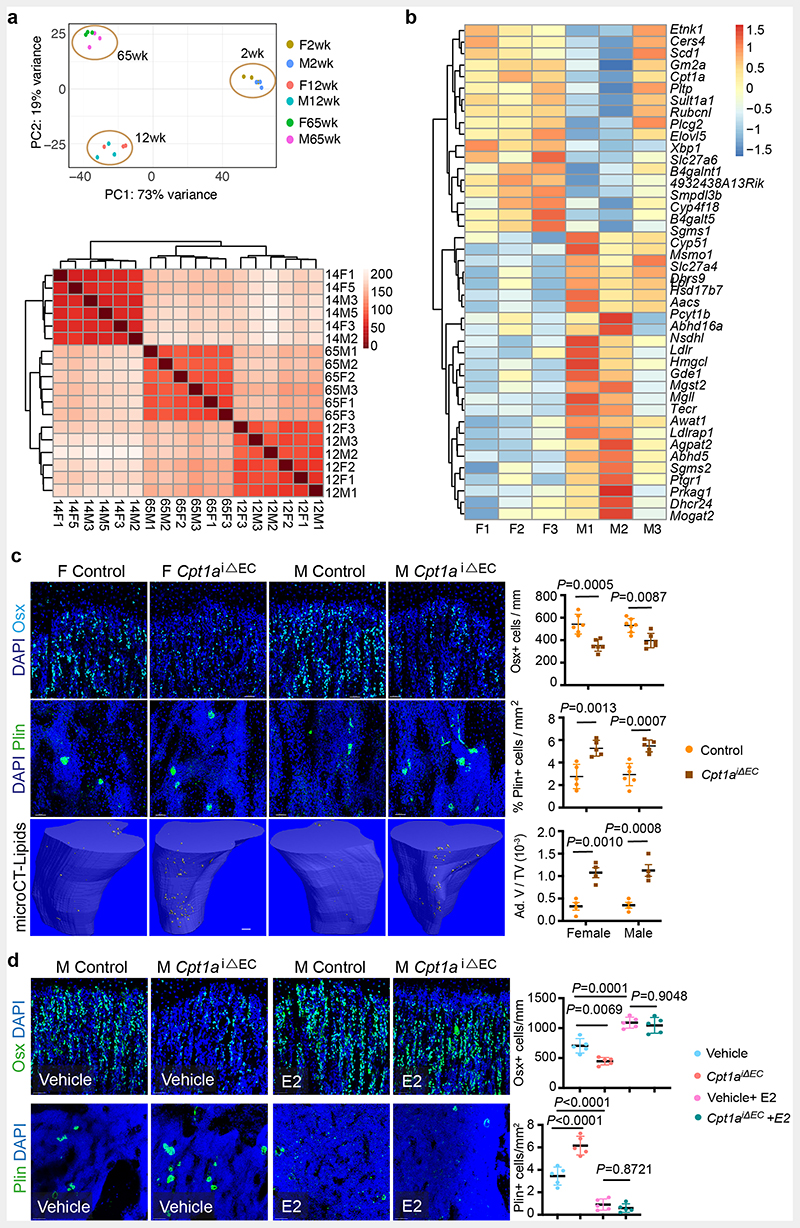
Lipid metabolism of BECs **a,** PCA plot of RNAseq data showing clustering of purified male and female BECs from 2 weeks, 12 weeks, and 65 weeks old mice (n=3). Sample correlation heatmap of gene expression pattern show clustering of age groups **b,** Heatmap showing genes associated with fatty acid metabolism, which are differentially expressed in aged mouse BECs between males and females **c,** Representative confocal images showing cell types within the BM microenvironment of *Cpt1a* mutants with graphs showing quantification of Osterix+ cells per mm of mp (Control n=6F, n=7M; Mutant n=6F, n=6M); Perilipin+ area per mm^2^ area (Control n=5F, n=6M; Mutant n=5F, n=5M). Representative microCT images with quantification show an increase in BM adipocyte volume in *Cpt1a* mutants (n=4). Data are mean ± s.d for image quantification and mean ± s.e.m for microCT data; Two-Way ANOVA with Tukey’s test. Scale bars 100μm (microCT), 40μm (confocal) **d,** Representative confocal images show Osx+ cells and Perilipin+ adipocytes in male control and *Cpt1a* ^iΔEC^ mutant mice treated with E2. Graphs show quantifications of Osx+ cells per mm of mp (n=5) and Perilipin+ cells per mm^2^ (n=5). Data are mean ± s.d.; Two-Way ANOVA with Tukey’s test. Scale bars 50μm

**Fig. 3:**
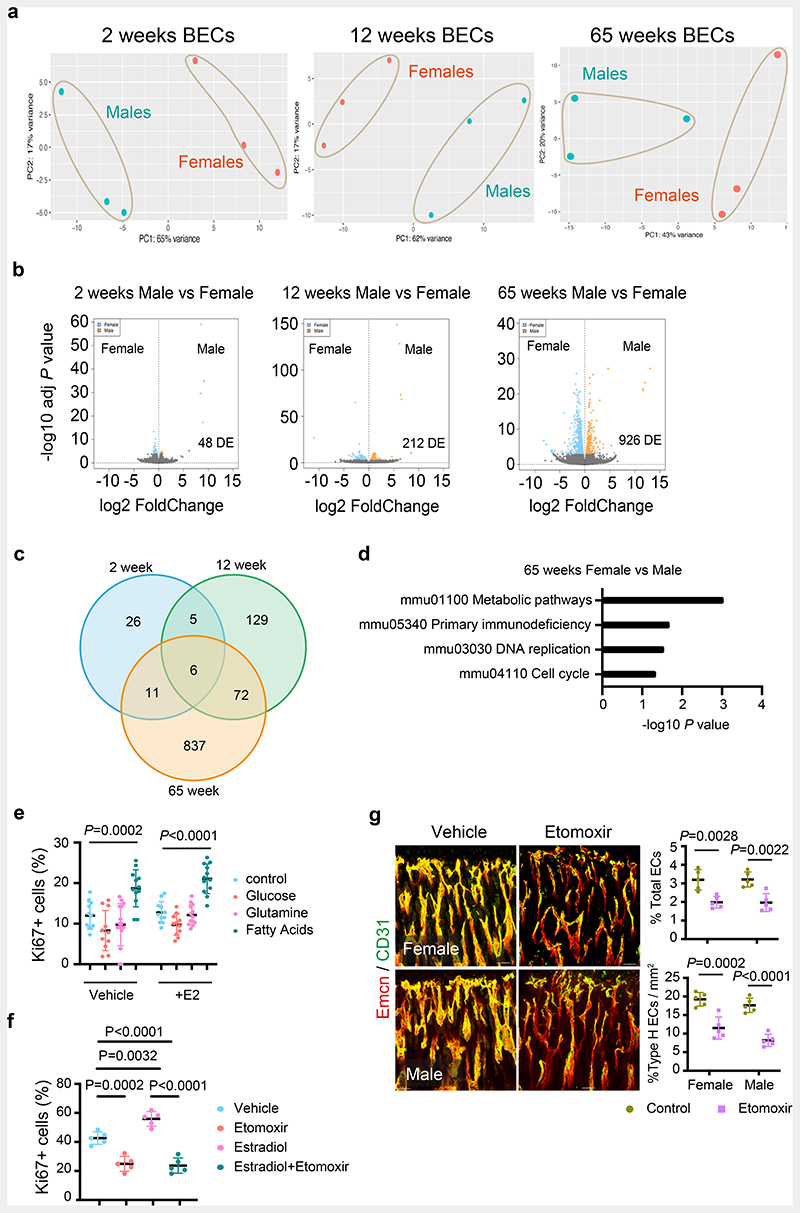
Metabolic regulation of BECs **a**, PCA plots of RNAseq data showing clustering by sexes in BECs from 2 weeks, 12 weeks, and 65 weeks old mice (n=3). **b**, Volcano plots show the number of significant FDR-corrected (Bonferroni) *P*-values (*P*_adj_<0.01) differentially expressed (DE) genes versus log2 fold change between female and male bone endothelial cells of 2 weeks, 12 weeks, and 65 weeks old mice. DE genes were obtained by fitting each gene to a generalized linear model followed by hypothesis testing using the Wald test. **c**, Venn diagram showing the overlap in differentially expressed genes between males and female bone endothelial cells of 2 weeks, 12 weeks, and 65 weeks old mice. **d**, Generally Applicable Gene-set Enrichment for Pathway Analysis (GAGE) for differentially expressed genes in RNAseq data showing biological processes that differ between aged male and female BECs. Gene data sets of metabolic pathways, cell cycle and DNA are upregulated, and primary immunodeficiency pathway genes are downregulated. The significance of enrichment was calculated using 2-sample *t-tests*, and the significant KEGG terms were selected using a *P*-value < 0.05. **e**, Graph showing quantification of Ki-67+ cultured BECs in the nutrient-free medium supplemented with glucose, glutamine or fatty acids, with vehicle (Control n=9, Glucose n=11, Glutamine n=15, Fatty acids n=12) and E2 treatment (Control n=11, Glucose n=11, Glutamine n=13, Fatty acids n=12); data are mean ± s.d; Two-Way ANOVA with Tukey’s test **f**, Graph showing quantification of Ki-67+ cultured BECs treated with Vehicle or E2, with or without Etomoxir treatment (n=5); data are mean ± s.d; Two-Way ANOVA with Tukey’s test **g**, Representative confocal images of Type-H (Emcn^high^ CD31^high^) vessels in Vehicle and Etomoxir-treated mice; quantification of type-H ECs normalised to the area of the metaphysis (mp) (n=5); Data are mean ± s.d.; Unpaired two-tailed t-test. Total ECs (CD31+ CD45-Ter119-) quantified by flow cytometry (n=5); Data are mean ± s.e.m.; Unpaired two-tailed t-test. Scale bars 50μm.

Oestrogen-mediated changes in the BM and similar endothelial ER mutant phenotypes in both female and male mice intrigued us to investigate sex differences in BECs. We performed RNA-seq analysis for purified BECs of 2-, 12- and 65-week old female and male mice to understand their sex differences. Gene expression analysis at different stages of growth and ageing would provide age-related changes in endothelial sex differences. Principal Component Analysis (PCA) separated male and female ECs into two clusters indicating variation between sexes at all age groups (Fig. 3a and Supplementary Dataset 1). The sex differences in BEC transcriptomes increased by age, with 48 differentially expressed (DE) genes in 2-weeks mice compared to 212 and 926 in 12- and 65-weeks respectively at *P_adj_<0.01* significance level (Fig. 3b). We observed a strikingly low overlap in DE genes between the age groups with the highest overlap of 72 genes were detected between adult and aged mice (Fig. 3c and Extended Data Fig. 6a). To understand the significance of the gene expression data, we performed Generally Applicable Gene-set Enrichment for Pathway Analysis (GAGE) for DE genes of 65-weeks data^19^. A total of four gene sets displayed significant levels of differences with a conspicuous enrichment of metabolic pathway gene sets (Fig. 3d). Analysis of differentially expressed genes (*P_adj_*<0.05) in this metabolic pathway gene sets showed a total of 142 genes altered between male and female BECs with 43 genes being associated with fatty acid metabolism (Extended Data Fig. 6b and Supplementary Dataset 1). However, there is limited understanding of BEC's metabolism and its role in angiogenesis.

**Fig. 4:**
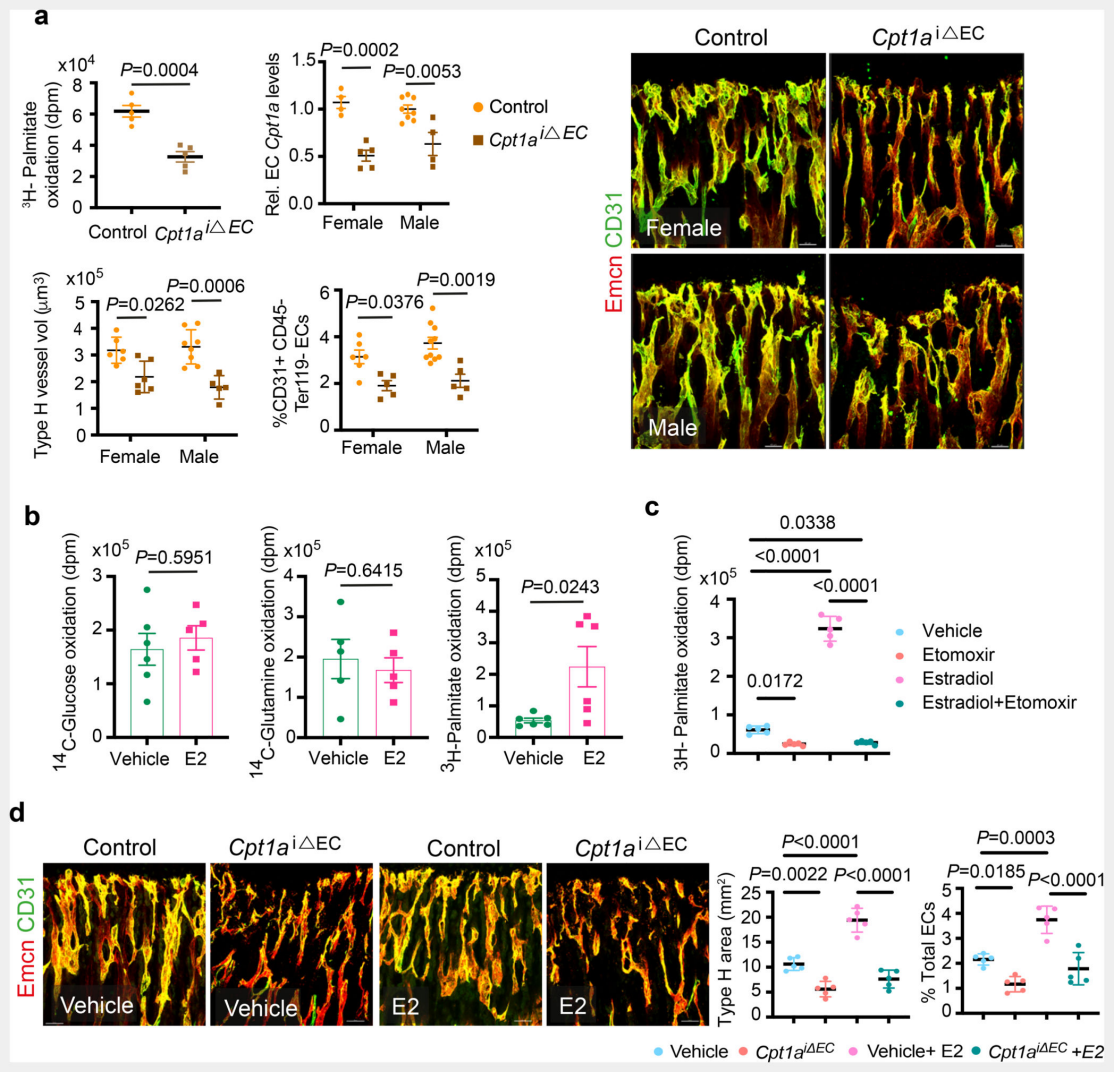
Oestrogen-mediated bone endothelial growth is supported by fatty acids **a,** Graph showing oxidation of ^3^H-labelled Palmitate by control and *Cpt1a* deleted BECs. Graph shows *Cpt1a* mRNA levels in purified BECs from control and *Cpt1a* ^iΔEC^ mutant bones. Representative confocal images of Type-H (Emcn^high^ CD31^high^) vessels in control and endothelial *Cpt1a* deletion mutants, with quantification of type-H blood vessel volume (Control n=6F, n=8M; Mutant n=6F, n=5M) and total ECs (CD31+ CD45-Ter119-) characterised by flow cytometry (Control n=6F, n=5M; Mutant n=10F, n=5M); Data are mean ± s.d. for image quantification (type-H volume) and mean ± s.e.m for H-Palmitate, flow cytometry and qPCR data; Two-Way ANOVA with Tukey’s test performed except ^3^H-Palmitate data (Unpaired two-tailed t-test). Scale bars 40μm. **b,** Graphs showing Glucose (Vehicle n=6, E2 n=5), Glutamine (Vehicle n=5, E2 n=5) and Palmitate (Vehicle n=6, E2 n=6) oxidation in Vehicle and E2-treated BECs, measured by radioactivity (disintegrations per minute, dpm). Data are mean ± s.e.m.; Unpaired two-tailed t-test **c**, Graph showing palmitate oxidation by BECs treated with Vehicle or E2, with or without Etomoxir treatment (n=5); data are mean ± s.e.m; Two-Way ANOVA with Tukey’s test **d,** Representative confocal images depicting Type-H (Emcn^high^ CD31^high^) vessels in control and *Cpt1a* ^iΔEC^ mutant mice treated with E2. Scale bars 50μm. Quantifications show Type-H vessel ECs normalised to the area of mp (Control n=5F, n=5M; Mutant n=5F, n=5M) and Total ECs (CD31+ CD45-Ter119-) quantified by flow cytometry (Control n=5F, n=5M; Mutant n=5F, n=5M). Data are mean ± s.d. for image quantification and mean ± s.e.m for flow cytometry data; Two-Way ANOVA with Tukey’s test

To understand the basal metabolic regulation of BECs, we tested major metabolic pathways that are known to regulate angiogenesis in other systems ^20–22^. We checked for the nutrient supplements that could support the cultured BECs in the absence of fatty acids (serum-free), glucose and glutamine. We found that supplementation of primary BECs with fatty acids (FA) but not glucose or glutamine sustained BECs *in vitro* (Fig. 3e). The result prompted us to further investigate and inhibit FA metabolism in BECs. Cpt1a, carnitine palmitoyltransferase 1a, is a rate-limiting enzyme of FA oxidation by transporting FAs into mitochondria. Etomoxir, Cpt1a inhibitor treatment reduced the growth of cultured BECs (Fig. 3f). Similarly, administration of etomoxir in P10 mice for 10 days reduced the growth of blood vessels in bones (Fig. 3g). To investigate the bone endothelial-specific role of FA metabolism, we genetically targeted Cpt1a in ECs using *Cpt1a floxed* mice ^21^ bred with EC-specific *Cdh5(PAC)CreERT2* ^9, 10, 17^. Loss of Cpt1a function in BECs showed reduced oxidation of radiolabelled ^3^H-Palmitate, indicating impaired FA metabolism (Fig. 4a). Analysis of blood vessels showed a reduction in total and type-H ECs of *Cpt1a* ^iΔEC^ bones compared to their littermate controls indicating defective blood vessel growth (Fig. 4a). In addition, *Cpt1a* ^iΔEC^ mutant bones showed reduced OBLs and increased adipocytes. Micro-CT analysis of mutant bones confirmed the changes in bone and lipid contents (Extended Data Fig. 6c), suggesting the importance of endothelial FA metabolism in regulating the BM.

We next investigated how oestrogen influenced endothelial metabolism by treating primary BECs with E2. E2 did not alter the FA dependency of BECs in nutrient-depleted conditions (Fig. 3e). Further, increased oxidation of ^3^H-Palmitate compared to unaltered oxidation of ^14^C-Glucose or ^14^C-Glutamine suggested that E2 treatment promoted the use of FAs (Fig. 4b). Notably, etomoxir inhibited the E2-mediated cellular proliferation (Fig. 3f) and FA oxidation (Fig. 4c). To understand *in vivo* relation, we administered E2 in *Cpt1a* ^iΔEC^ mutant mice to investigate angiogenesis. Administration of E2 did not recover angiogenesis inhibited in *Cpt1a* ^iΔEC^ mutant bones, supporting the downstream FA dependency of E2-mediated blood vessel growth (Fig. 4d), while E2 increased OBLs and reduced adipocytes in the mesenchymal compartment (Extended Data Fig. 6d). Together, the data indicated that oestrogen required fatty acids to drive angiogenesis in bones.

## Oestrogen promotes FA uptake in BECs

**Extended Data Fig. 7:**
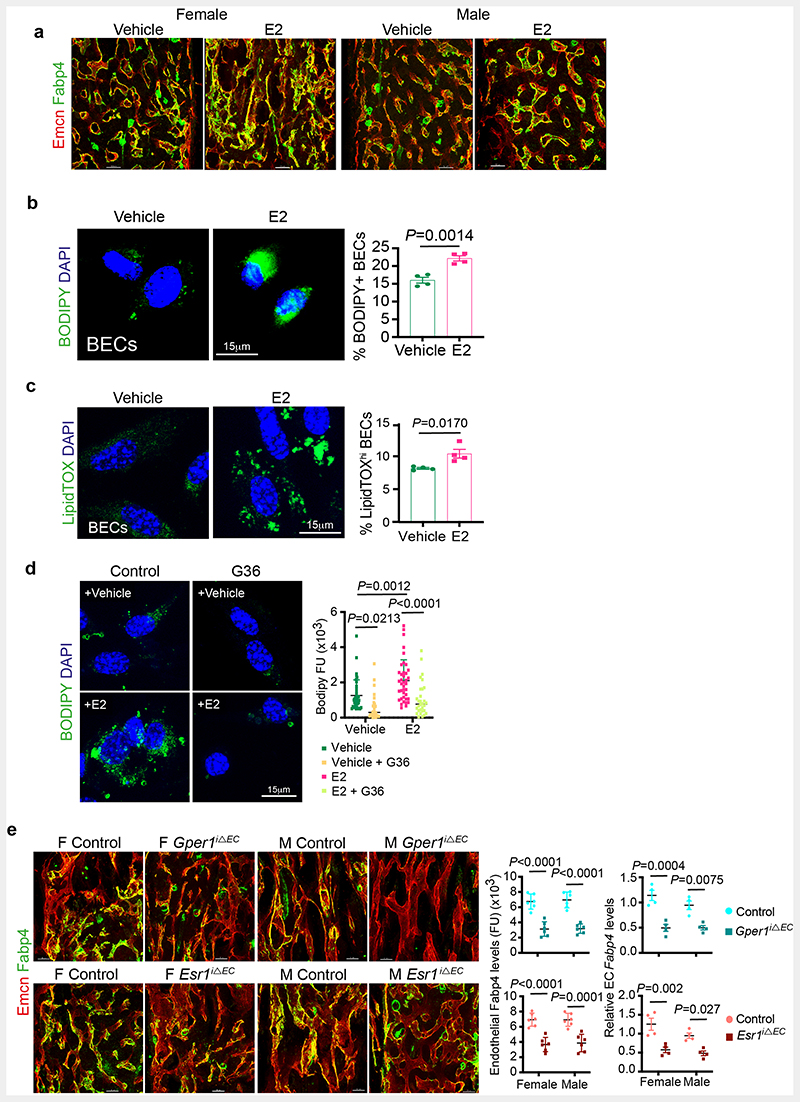
Lipid transport mechanism in BECs **a**, Immunostainings show Fabp4 expression (green) in blood vessels (Emcn, red) of vehicle and E2 treated tibia of both male and female mice (n=7). Scale bars 50μm **b**, Representative confocal images of lipid uptake of BODIPY-conjugated synthetic FAs in Vehicle and E2-treated BECs (n=4), with quantification by flow cytometry indicating an increase in lipid uptake with E2 treatment. Data are mean ± s.e.m.; Unpaired two-tailed t-test. Scale bars 15 μm **c**, Representative confocal images of intracellular lipid levels in Vehicle and E2-treated BECs. The graph shows flow cytometry quantification of BECs having high LipidTOX (n=4) levels in vehicle and E2 conditions. Data are mean ± s.e.m.; Unpaired two-tailed t-test. Scale bars 15μm **d**, Representative confocal images of lipid uptake of BODIPY-conjugated synthetic FAs in Vehicle and E2-treated BECs upon *Gper1* inhibitor G36 treatment. Quantification shows reduced fluorescence units of endothelial BODIPY upon *Gper1* inhibition, across multiple 63x fields of view (Vehicle n= 37, Vehicle+G36 n= 30; E2 n= 39, E2+G36 n= 30). Data are mean ± s.d.; Two-Way ANOVA with Tukey’s test. Scale bars 15μm **e**, Representative confocal images and quantification of endothelial Fabp4 levels in mice with oestrogen-receptor deletions *Esr1* (Control n=6F, n=6M; Mutant n=6F, n=6M) and *Gper1* (Control n=6F, n=6M; Mutant n=6F, n=6M). Data are mean ± s.d.; Two-Way ANOVA with Tukey’s test. Relative *Fabp4* transcript levels were also quantified in purified endothelial cells from endothelial Gper1 (n=4) and Esr1 (n=4) mutants compared to their littermate control. Data are mean ± s.e.m; Two-Way ANOVA with Tukey’s test. Scale bars 50μm

**Fig. 5:**
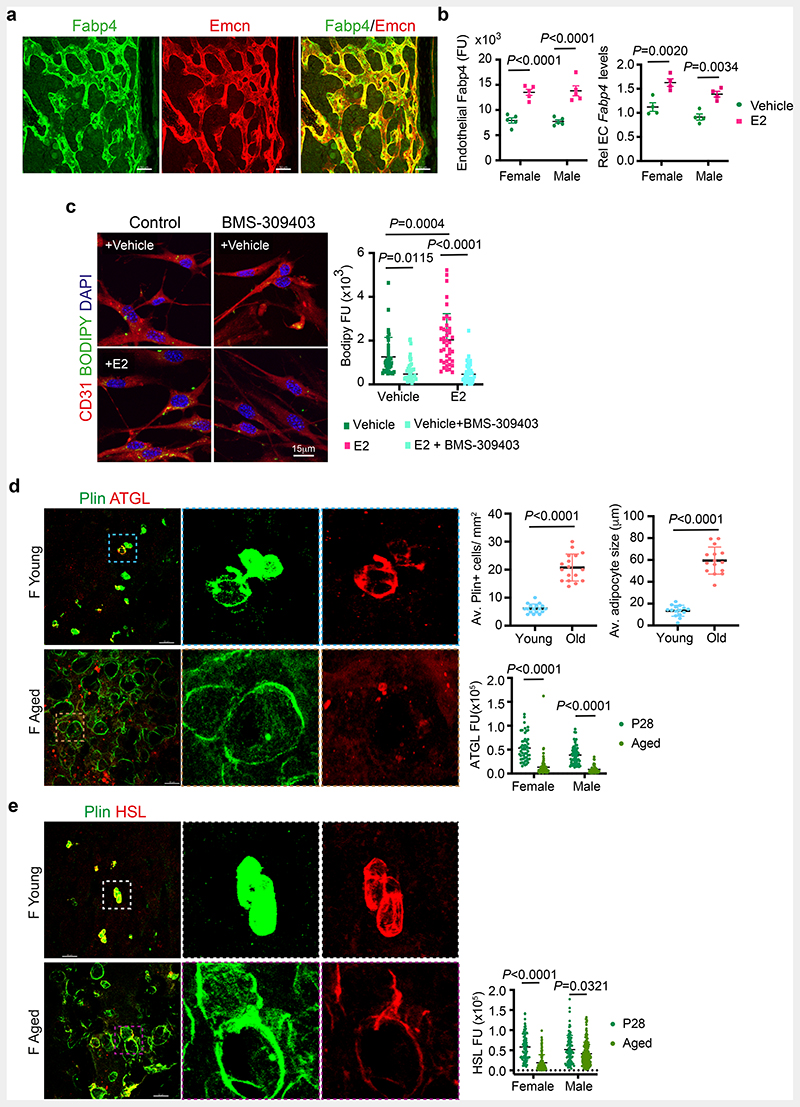
Oestrogen regulation of endothelial lipid transport **a**, Maximum intensity projection of confocal image showing overlap of Fabp4 (green) expression in bone endothelial cells overlap with the Endomucin (Emcn, red) expression. Scale bars 50μm. **b**, Graphs show quantification of Fabp4 protein levels observed in bone tissues sections of vehicle and E2 treated bones (n=5) imaged using confocal microscopy. Data are mean ± s.d.; Two-Way ANOVA with Tukey’s test. Transcript levels were quantified from isolated bone endothelial cells of vehicle and E2 (n=5) administered mice using quantitative PCR. Data are mean ± s.e.m.; Two-Way ANOVA with Tukey’s test **c**, Representative confocal images of lipid uptake of BODIPY-conjugated synthetic FAs in Vehicle and E2-treated BECs upon Fabp4 inhibitor BMS-309403 treatment. Scale bars 15μm. Quantification shows reduced fluorescence units (FU, intensity) of endothelial BODIPY upon Fabp4 inhibition across multiple 63x fields of view (Vehicle n= 37, Vehicle+BMS-309403 n= 36; E2 n= 41, E2+BMS-309403 n= 42) Data are mean ± s.d.; TwoWay ANOVA with Tukey’s test **d**, Representative confocal images of BM adipocytes (Perilipin+, green) from young and aged bones. Scale bars 50μm. Graphs show average Perilipin+ adipocytes number (n=17), size (n=15) and fluorescence intensity quantification indicating a decrease in lipolytic enzyme ATGL in aged (n= 84F,52M) compared to young mice (n= 50F,58M) per cell across multiple 20x fields of view. Data are mean ± s.d.; Two-Way ANOVA with Tukey’s test **e**, Representative confocal images of BM adipocytes (Perilipin+), with fluorescence intensity quantification indicating a decrease in lipolytic enzyme HSL in aged mice (HSL n= 120F,168M) compared to P28 (HSL n=78F,88M) per cell across multiple 20x fields of view. Data are mean ± s.d.; Two-Way ANOVA with Tukey’s test. Scale bars 50μm. F, female; M, male; Plin, perilipin.

Bone marrow adipocytes were suggested to release free fatty acids as an energy source for haematopoietic cells ^23^. We identified that BECs expressed Fatty Acid Binding Protein 4 (FABP4), a membrane lipid transporter involved in FA uptake, transport and metabolism (Fig. 5a). E2-treated bones showed upregulation of FABP4 expression in their blood vessels (Fig. 5b and Extended Data Fig. 7a), which led us to investigate lipid transport in BECs. To analyse FA uptake in BECs, we provided a short 30 minutes pulse of BODIPY-conjugated synthetic FAs in the medium. Detection of high BODIPY levels in E2 treated BECs compared to controls indicated the promotion of FA uptake by E2 (Fig. 5c and Extended Data Fig. 7b). We also checked FA levels in BECs after E2 treatment using LipidTOX. Detection of more BECs with high intracellular lipid levels supported lipid uptake upon E2 treatment (Extended Data Fig. 7c). To understand the functional role of FABP4 in FA uptake, we treated BECs with pharmacological FABP4 inhibitor, BMS-309403 in the presence and absence of E2. Reduced BODIPY levels in BECs treated with BMS-309403 (Fig. 5c) indicated the involvement of FABP4 in E2-mediated FA uptake. Further, we investigated lipid uptake in the presence of ER signalling inhibitor G36, a pharmacological antagonist of Gper1. Impaired BODIPY uptake in BECs treated with G36 confirmed lipid transport as a downstream mechanism of ER signalling (Extended Data Fig. 7d). Further, reduced FABP4 expression in blood vessels of *Esr1*^iΔEC^ and *Gper1* ^iΔEC^ mutant mice (Extended Data Fig. 7e) indicated the association between defective FA uptake with reduced angiogenesis.

## Metabolic coupling of BECs and adipocytes

Defective FA uptake and angiogenesis in BECs of *Esr1*^iΔEC^, and *Gper1* ^iΔEC^ mutant bones were associated with the accumulation of adipocytes in the microenvironment. Age-related decline in angiogenesis and osteogenesis ^6, 7, 9, 24, 25^ involved a concurrent increase in adipocytes ^26, 27^. Reduced angiogenic status of ageing bones is associated with an increase in the number and size of adipocytes in the BM. On the contrary, young bones with high angiogenesis showed smaller and fewer adipocytes, indicating an inverse relationship between angiogenic ECs and adipocytes (Fig. 5d and e). Smaller adipocytes present in young bones showed high expression of lipolytic enzymes, Adipose triglyceride lipase (ATGL) and Hormone Sensitive Lipase (HSL) compared to larger adipocytes present in aged bones. Thus, limited adipocytes present in young angiogenic bones displayed high levels of lipolytic enzymes. Likewise, aged bones with reduced angiogenesis showed low levels of lipolytic lipases in their adipocytes (Fig. 5d and e).

**Extended Data Fig. 8:**
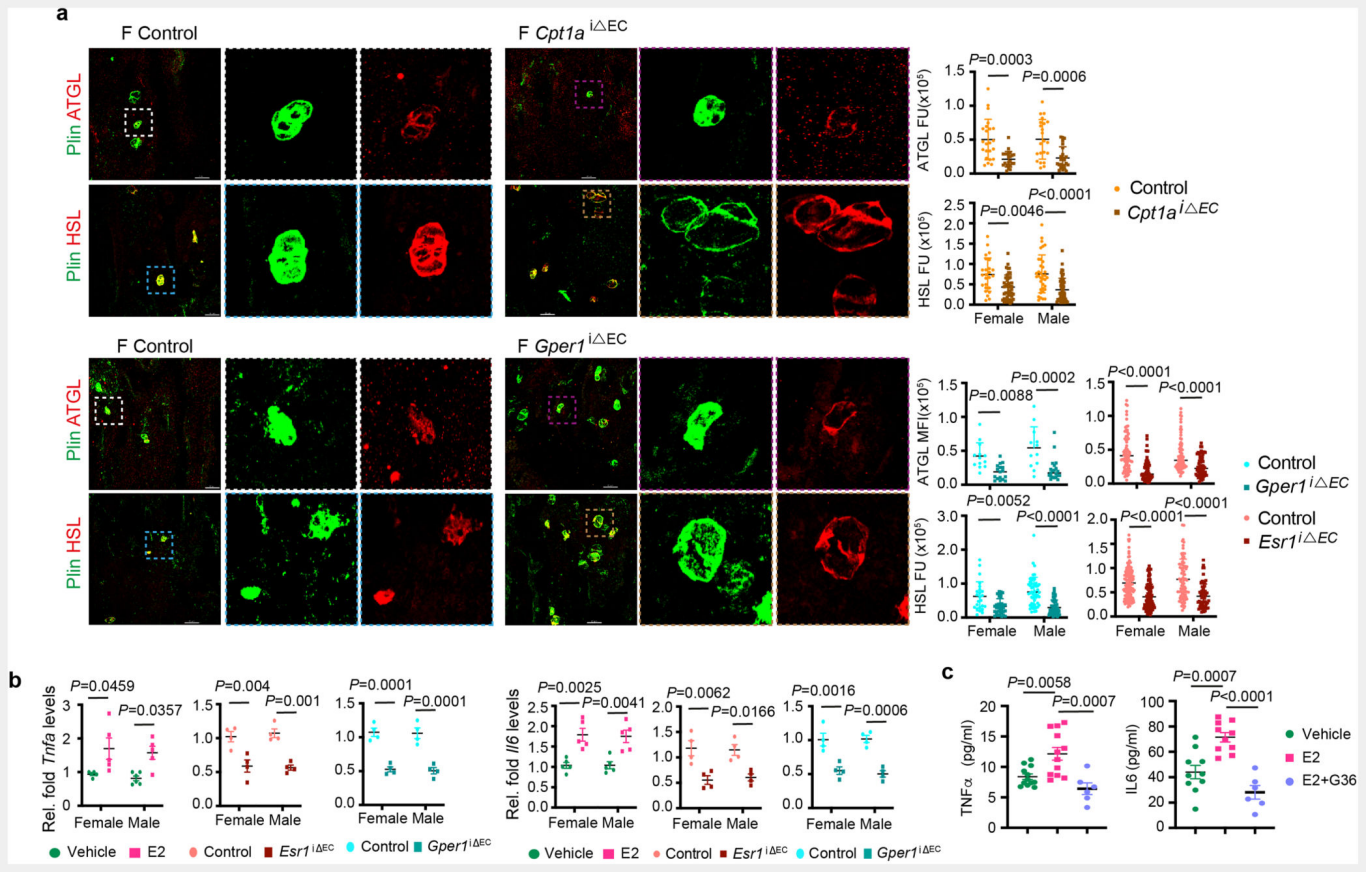
Metabolic coupling of BECs with adipocytes **a**, Representative confocal images of BM adipocytes (Perilipin+ cells) and lipolytic enzymes (ATGL and HSL) in *Cpt1a* and *Gper1* mutant bones and their respective littermates. Scale bars 50μm. Graphs show with fluorescence quantifications indicating a decrease in lipolytic enzymes (ATGL and HSL) in *Cpt1a* (ATGL: Control n=23F, 15M; Mutant n=23F, 24M. HSL Control n=19F,36M; Mutant n=42F,54M), *Gper1* (ATGL: Control n=13F,12M; Mutant n=17F, 21M. HSL: Control n=19F,50M; Mutant n=37F,49M) and *Esr1* (ATGL: Control n=75F,72M; Mutant n=63F, 62M. HSL: Control n=127F,79M; Mutant n=106F,60M) deletion, per cell across multiple 20x fields of view. Data are mean ± s.d.; Two-Way ANOVA with Tukey’s test **b**, Graphs showing relative transcript levels of *Tnfa* and *Il6* mRNA in isolated BECs of E2-treated mice (Vehicle n=5F, n=5M; E2 n=5F, n=5M), and endothelial *Cpt1a* and *Gper1* deletion mutants (control n=4F, n=4M; mutant n=4F, n=4M). Data are mean ± s.e.m.; Two-Way ANOVA with Tukey’s test **c**, Graph showing increased TNFα (vehicle n=12, E2 n=12, E2+ G36 n=6) and IL-6 (vehicle n=12, E2 n=12, E2+ G36 n=6) levels quantified from cell culture supernatants of BECs treated with vehicle, E2 and E2+ G36. Data are mean ± s.e.m.; One-Way ANOVA with Tukey’s test

Adipocytes have previously been shown to express oestrogen receptors and respond to circulating oestrogen levels ^28, 29^. However, variation of adipocyte numbers in endothelial mutants (see adipocyte phenotypes of *Esr1*^iΔEC^, *Gper1* ^iΔEC^ and *Cpt1a* ^iΔEC^ mice) prompted us to investigate the expression of lipolytic enzymes in these mutants. Adipocytes present in *Cpt1a* ^iΔEC^ and *Gper1* ^iΔEC^ mutant bones showed low levels of lipolytic lipases (Extended Data Fig. 8a). The data indicated the involvement of BECs in regulating lipolysis of adipocytes in the BM. To identify potential EC-derived signals acting on adipocytes, isolated BECs were analysed for the expression of lipolytic factors. Purified BECs from E2 administered mice showed upregulation of *Tnfa* and *Il6*, well-recognised paracrine lipolytic factors. Impaired oestrogen receptor signalling in *Esr1* ^iΔEC^ and *Gper1* ^iΔEC^ mutant BECs showed downregulation of endothelial *Tnfa* and *Il6* levels (Extended Data Fig. 8b). Reduced expression of these lipolytic factors supported the accumulation of adipocytes in these mutant bones. Likewise, E2 treatment of *in vitro* cultured BECs released higher levels of TNF-α and IL-6 in the medium compared to control treatment (Extended Data Fig. 8c), confirming their angiocrine release from BECs. Decreased angiogenesis and thus lipid use in BECs is associated with the reduced lipolysis and accumulation of adipocytes in the BM. These data suggest metabolic interaction between angiogenesis and lipolysis in the bone microenvironment.

## Accumulation of lipid peroxides in ageing BECs

**Extended Data Fig. 9:**
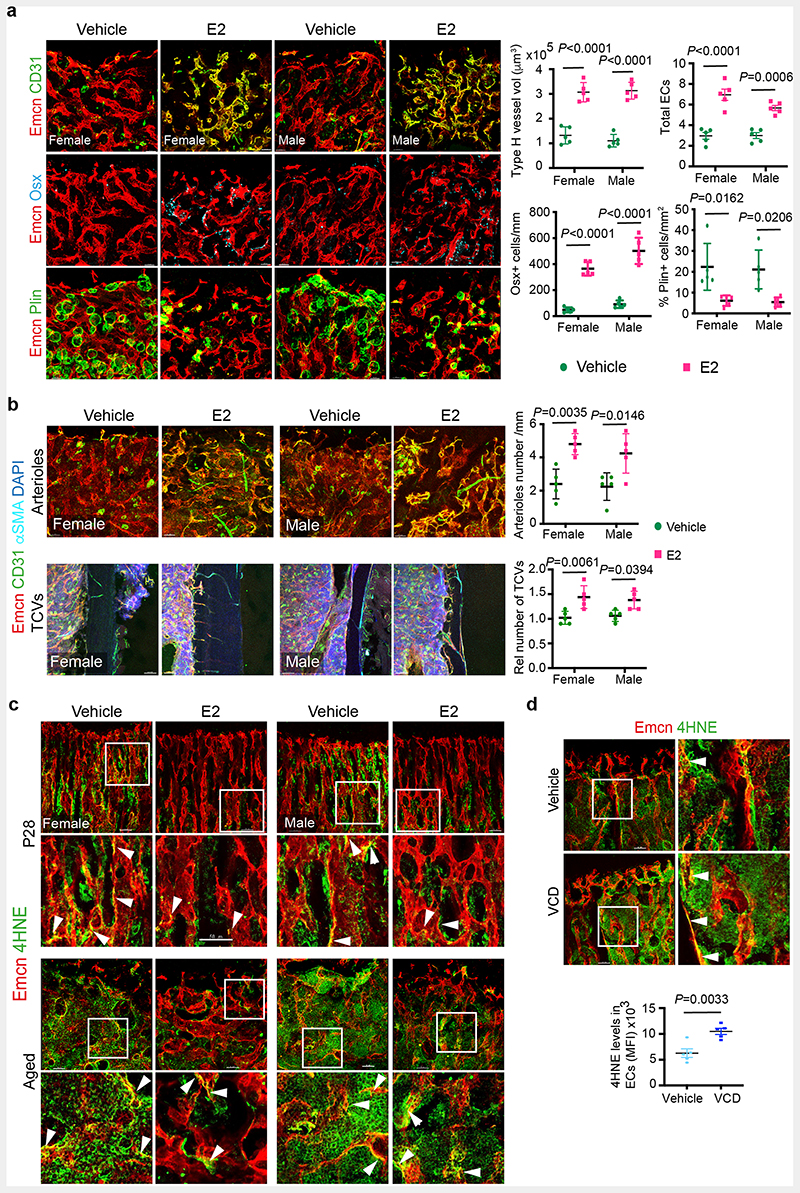
Oestrogen recovers age-related bone loss **a**, Representative confocal images show Type-H vessels (yellow, Emcn^high^ CD31^high^), OBLs (Osterix+) and BM adipocytes (Perilipin+) in aged mice treated with E2 (Vehicle n=5F, n=5M; E2 n=5F, n=5M). Scale bars 40μm. Graphs show quantification of total ECs, type-H vessels, Osx+ and Perilipin+ cells in bones of vehicle and E2 treated mice. Data are mean ± s.e.m. for flow cytometry data and mean ± s.d. for image quantification, Two-Way ANOVA with Tukey’s test **b**, Confocal images of bone sections immunostained for CD31 (green), endomucin (red), alpha-smooth muscle actin (αSMA, cyan) show distribution of CD31+ Emcn-arterioles and TCVs in aged mice treated with Vehicle and E2. Quantification graphs show an increase in arterioles and TCVs (n=5) after E2 administration in mice; Data are mean ± s.d.; Two-way ANOVA with Tukey’s test. Scale bars 50μm (arterioles), 100μm (TCVs) **c**, Representative confocal images of 4HNE levels in bone endothelium of vehicle and E2-treated young (P28; n=8) and aged (>60 weeks; n=4) mice. Images show inset depicting endothelial expression of 4HNE illustrated by arrowheads. Scale bars 50μm **d**, VCD menopause model with insets indicating endothelial expression of 4HNE illustrated by arrowheads (Vehicle n=5; VCD n=5). Data are mean ± s.d.; Unpaired two-tailed t-test. Scale bars 50μm

**Extended Data Fig. 10:**
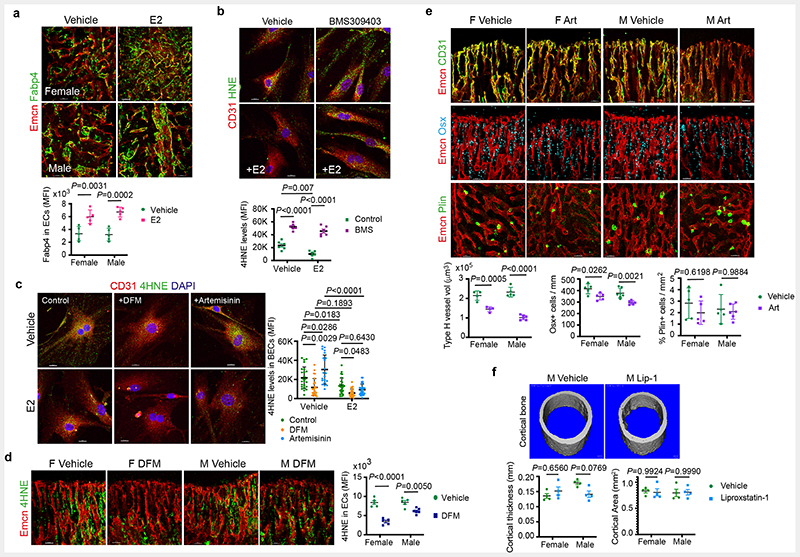
Oestrogen recovers age-related bone loss **a**, Representative confocal images of endothelial Fabp4 expression in aged mice (n=5), showing an increase after E2 treatment. Data are mean ± s.d.; Two-Way ANOVA with Tukey’s test. Scale bars 50μm **b**, Representative confocal images with quantification of 4HNE levels in bone endothelial cells treated with vehicle and FABP4 inhibitor BMS 309403 in the presence of E2. Quantification showed high 4HNE levels upon Fabp4 inhibition, which was not recovered by E2 treatment (n= 6). Data are mean ± s.d.; Two-Way ANOVA with Tukey’s test. Scale bars 15μm **c**, Representative confocal images of BECs treated with Control, Deferoxamine (DFM) or Artemisinin (Art). Graph shows quantification of fluorescence intensity of endothelial 4HNE levels (Vehicle n= 24, E2 n=26; Vehicle+DFM n=26; E2+DFM n=27; Vehicle+Art n=21; E2+Art n=23) Data are mean ± s.d.; Two-Way ANOVA with Tukey’s test. Scale bars 15μm **d**, Representative confocal images with quantification of 4HNE levels in bone endothelium of vehicle (n=5F, n=5M) and DFM-treated mice (n=5F, n=6M) at P28. Data are mean ± s.d.; Two-Way ANOVA with Tukey’s test. Scale bars 50μm **e**, Confocal images show type-H vessels, OBLs and perilipin expression in BM microenvironments of mice treated with Artemisinin (Art). Graphs showing quantification of Type-H vessel volume per field of mp (Vehicle n=4F, n=5M; Artemisinin n=4F, n=6M), Osterix+ cells per mm of mp (Vehicle n=5F, n=7M; Artemisinin n=5F, n=6M), and Perilipin+ area per mp area (Vehicle n=5F, n=5M; Artemisinin n=5F, n=6M). Data are mean ± s.d. Two-Way ANOVA with Tukey’s test. Scale bars 50μm **f**, Representative microCT 3D-rendered images show cortical bones of aged male mice treated with vehicle and Liproxstatin-1 (Lip-1). Graphs show cortical bone thickness (in mm) and cortical bone area of aged male and female mice treated with vehicle (n=4F, 4M) and Liproxstatin-1 (n=4F, 4M). Data are mean ± s.e.m.; Two-Way ANOVA with Tukey’s test. Scale bars 100μm

**Fig. 6:**
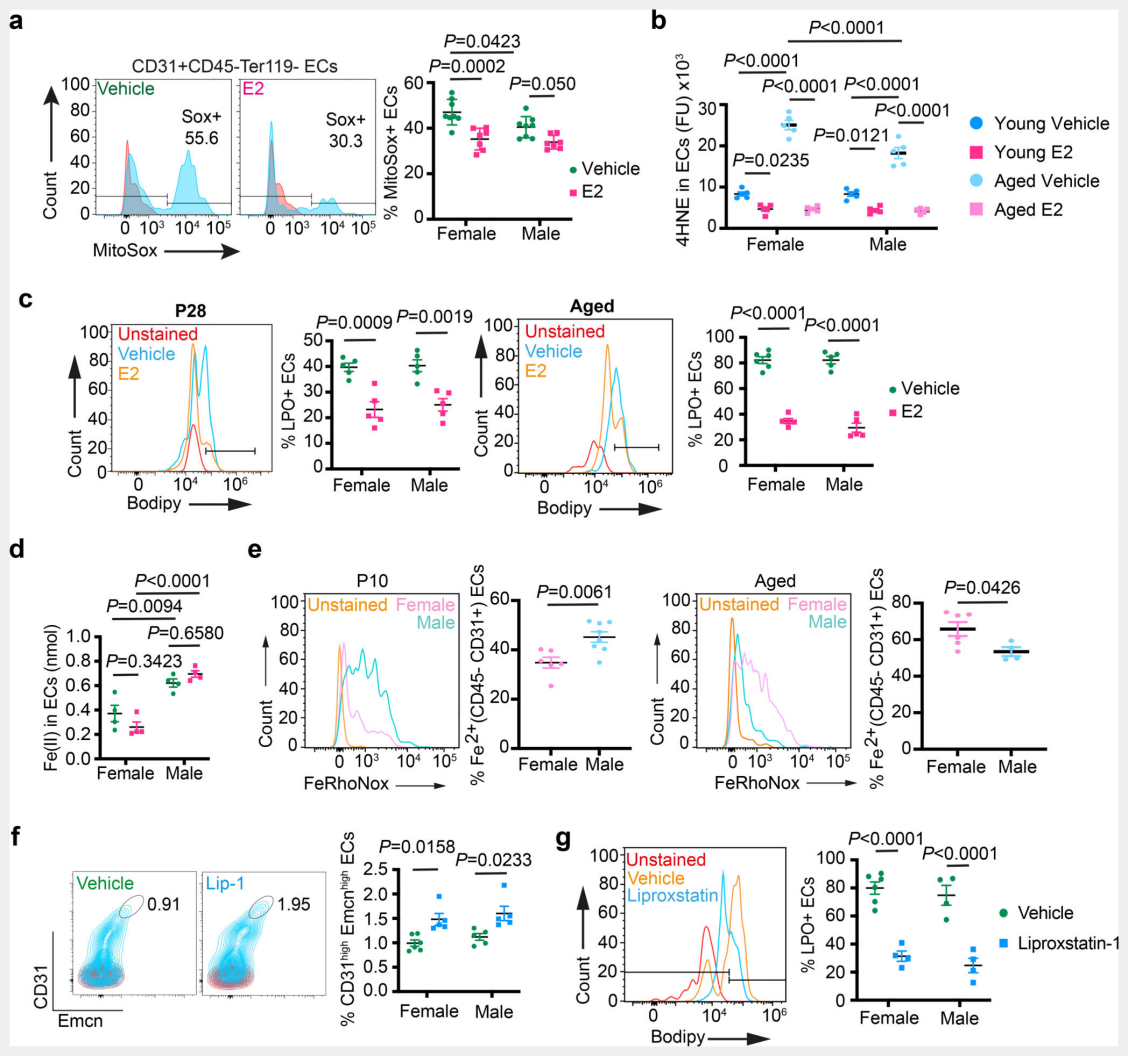
Defective lipid metabolism promotes vascular ageing in bones **a**, Representative flow cytometry plots of ROS generation in ECs from Vehicle (n=8F, n=8M) and E2-treated (n=7F, n=7M) mice. Red peaks in the histogram plots indicate unstained cells. Quantifications show a reduction in ROS upon E2 treatment. Data are mean ± s.e.m.; Two-Way ANOVA with Tukey’s test **b**, Quantifications of 4HNE levels in ECs from immunostaining in young Vehicle (n=5F, n=5M) and E2-treated (n=5F, n=5M) mice; and aged Vehicle (n=5F, n=5M) and E2-treated (n=5F, n=5M) mice showing a reduction in 4HNE levels upon E2-treatment. Data are mean ± s.d.; Two-Way ANOVA with Tukey’s test **c**, Representative flow cytometry plots of Lipid peroxide (LPO) generation in ECs from young Vehicle (n=5F, n=5M) and E2-treated (n=5F, n=5M), and aged Vehicle (n=6F, n=5M) and E2-treated (n=5F,n=5M) mice. Red peaks in the histogram plots indicate unstained cells. Quantifications show a reduction in LPO upon E2 treatment. Data are mean ± s.e.m.; Two-Way ANOVA with Tukey’s test **d**, Graph showing sex differences in intracellular ferrous ion concentrations in BECs of Vehicle and E2-treated mice (n=4). Data are mean ± s.e.m.; Two-Way ANOVA with Tukey’s test **e**, Representative flow cytometry plots with quantifications showing sex differences in intracellular ferrous ions in BECs at P10 (n=6F, n=8M) and aged (n=6F, n=4M) mice. Data are mean ± s.e.m.; Unpaired two-tailed t-test **f**, Representative flow cytometry plots with quantifications showing increase in Type-H ECs (CD31^high^ Emcn^high^) with Liproxstatin-1 (Lip-1) treatment in aged mice (Vehicle n=6F, n=5M; Lip-1 n=5F, n=5M). Data are mean ± s.e.m.; Two-Way ANOVA with Tukey’s test **g**, Representative flow cytometry plots of BEC populations of aged Vehicle and Lip-1-treated mice. Quantifications are of ECs with lipid ROS (Vehicle n=6F, n=4M; Lip-1 n=4F, n=4M), indicating a reduction upon Lip-1 treatment. Red peaks in the histogram plots indicate unstained cells. Data are mean ± s.e.m.; Two-Way ANOVA with Tukey’s test

Skeletal ageing is associated with changes in blood vessels that contribute to bone loss ^9, 24, 25^. Age-related decline in oestrogen levels ^30–33^ led to severe bone loss in females. Oestrogen administration has been shown to prevent age-related bone loss ^34–37^. Administration of E2 in ageing mice promoted total ECs, type-H capillaries and OBLs, and decreased adipocytes (Extended Data Fig. 9a). We also noted an increase in arterioles and transcortical vessels in the bones of E2 administered mice (Extended Data Fig. 9b). Age-associated increase in cellular reactive oxygen species (ROS)^38^ has been widely recognised. Remarkably, E2 administration in ageing mice significantly reduced endothelial ROS levels in blood vessels (Fig. 6a). Further analysis showed accumulation of lipid peroxides (LPO) in ageing BECs as evidenced using 4-Hydroxynonenal^39^ (HNE) levels and Bodipy-665/676, an LPO sensor. We found reduced LPO levels in BECs of E2-administered mice (Fig. 6b, c and Extended Data Fig. 9c). Likewise, an increase in endothelial HNE of menopause-induced bones argued for the high lipid peroxidation in oestrogen-depleted conditions (Extended Data Fig. 9d). FABP4 has been shown to contribute to protection against oxidative stress by reducing cellular (ROS) ^40^. Notably, E2 administration improved FABP4 expression in aged bone endothelium and indicated a potential relation between FABP4 and LPOs in BECs (Extended Data Fig. 10a). Inhibition of FABP4 in BECs showed accumulation of HNE, suggesting its involvement in LPO generation (Extended Data Fig. 10b). E2 supplementation could not reduce FABP4 mediated HNE accumulation indicating FABP4 dependency of E2 function (Extended Data Fig. 10c). In addition, the data argued for investigating E2 function in the regulation of multiple cellular mechanisms that result in the generation of peroxides.

## Ageing of bone endothelial cells

Cellular Fe^2+^ levels contribute to the generation of LPO in cells through a ferroptosis-dependent mechanism^41, 42^. To understand the contribution of Fe^2+^ in age-related increase in endothelial LPO levels, we investigated Fe^2+^ concentration in both male and female BECs. Intracellular labile Fe^2+^ concentration was higher in purified male BECs compared to females and was unaltered by E2 treatment in young BECs (Fig. 6d). We then used FeRhoNox-1 labelling to detect and quantify Fe^2+^ levels in BECs. FeRhoNox-1 labelling also detected high Fe^2+^ levels in young male BECs compared to females. Interestingly, quantification in aged bones showed a higher percentage of Fe^2+^ BECs in ageing females compared to males (Fig. 6e). Culturing of primary BECs with deferoxamine (DFM), an iron chelator resulted in the reduction of endothelial LPO levels. Correspondingly, induction of Fe^2+^ mediated peroxide production using artemisinin increased endothelial LPO levels. E2 treatment reduced LPO levels in both DFM and artemisinin-treated BECs *in vitro* (Extended Data Fig. 10c). DFM has been shown to promote type-H vessels and osteogenesis in aged mice ^9^. We found reduced LPO levels in BECs of mice administered with DFM (Extended Data Fig. 10d). Likewise, treatment of artemisinin (200mg/kg) in young mice led to a reduction in type-H capillaries and OBLs with no change in adipocytes (Extended Data Fig. 10e). This data suggested the role of Fe^2+^ in endothelial LPO production and thus ageing phenotype of blood vessels in the bone.

**Fig. 7:**
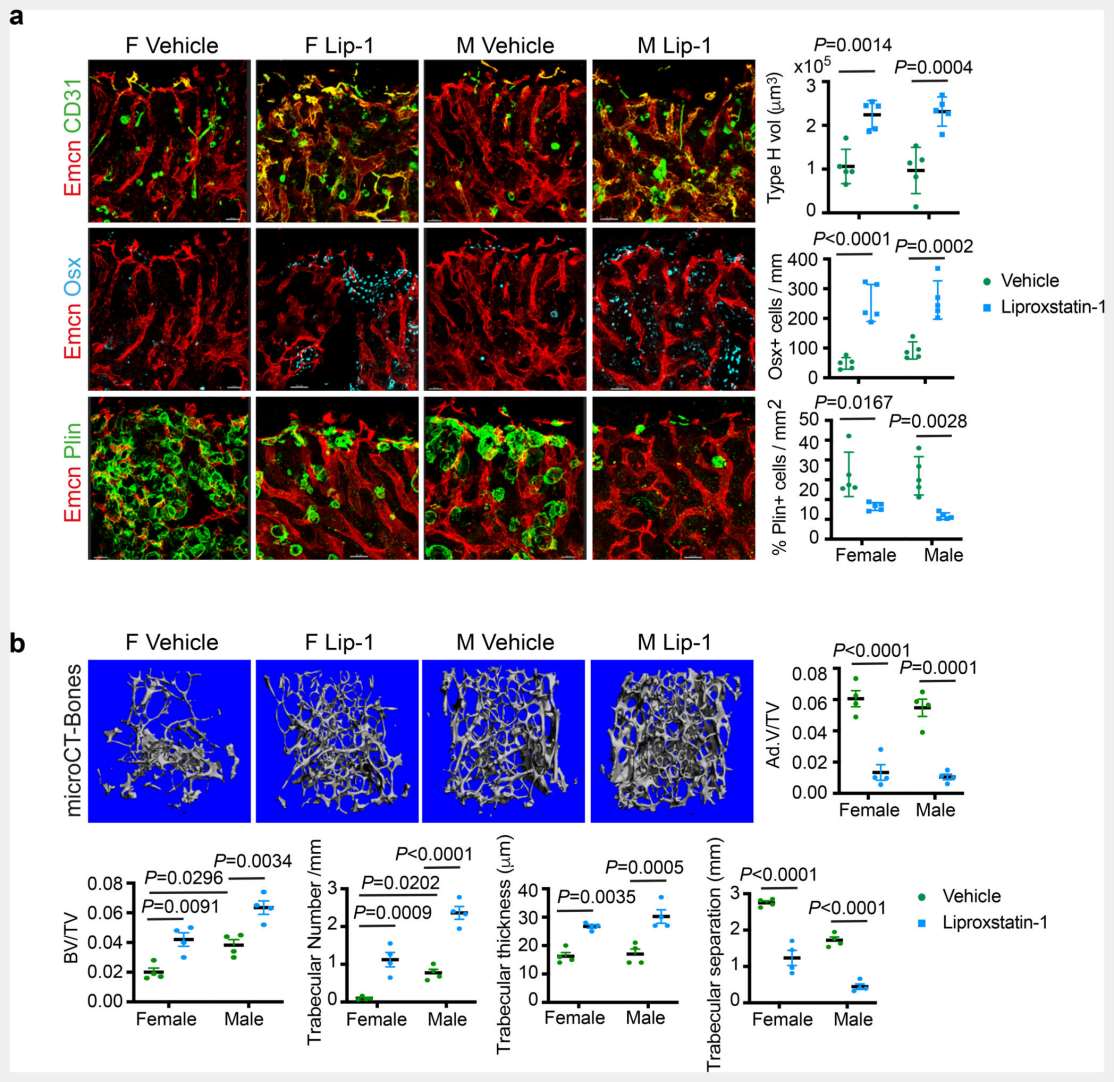
Liproxstatin-1 protects against age-related bone loss **a**, Representative confocal images of BM microenvironment in aged mice treated with Liproxstatin-1 (Lip-1), with graphs showing quantification of Type-H vessel volume per field of mp (Vehicle n=5F, n=5M; Liproxstatin-1 n=5F, n=5M), Osterix+ cells per mm of mp (Vehicle n=5F, n=5M; Liproxstatin-1 n=5F, n=5M), and Perilipin+ area per mm area (Vehicle n=5F, n=5M; Liproxstatin-1 n=5F, n=5M). Data are mean ± s.d.; Two-Way ANOVA with Tukey’s test. Scale bars: 40μm (vehicle), 50μm (Liproxstatin-1). **b**, Representative microCT images of bones from aged mice treated with Liproxstatin-1, with graphs showing quantification of trabecular bone volume, number, thickness and separation (n=4) and adipocyte volume (n=4). Data are mean ± s.e.m.; Two-Way ANOVA with Tukey’s test.

High basal Fe^2+^ levels in aged female ECs could aggravate LPO production and loss of type-H and bone in oestrogen-depleted conditions like menopause. The reduction of LPO production by E2 (Fig. 6b and c) suggested a potential mechanism underlying the clinical benefits of Hormone Replacement Therapy (HRT) for postmenopausal bone loss. To test this, we pharmacologically targeted LPO production using liproxstatin-1 (Lip-1), a lipid peroxide inhibitor in aged mice. Aged mice treated with vehicle control contained few type-H vessels compared to liproxstatin-1 (10mg/kg mouse) administered mice which showed an increase in angiogenic capillaries (Fig. 6f). Analysis of ROS levels in BECs demonstrated a reduction in liproxstatin-1 treated bones (Fig. 6g). Further, liproxstatin-1 mediated increase in type-H capillaries is associated with the increase in OBLs and reduction in adipocytes in aged bones (Fig. 7a). Micro-CT analysis of aged bones confirmed the increase observed in bone and reduction in lipid contents of liproxstatin-1 treated mice (Fig. 7b and Extended Data Fig. 10f).

## Discussion

**Fig. 8:**
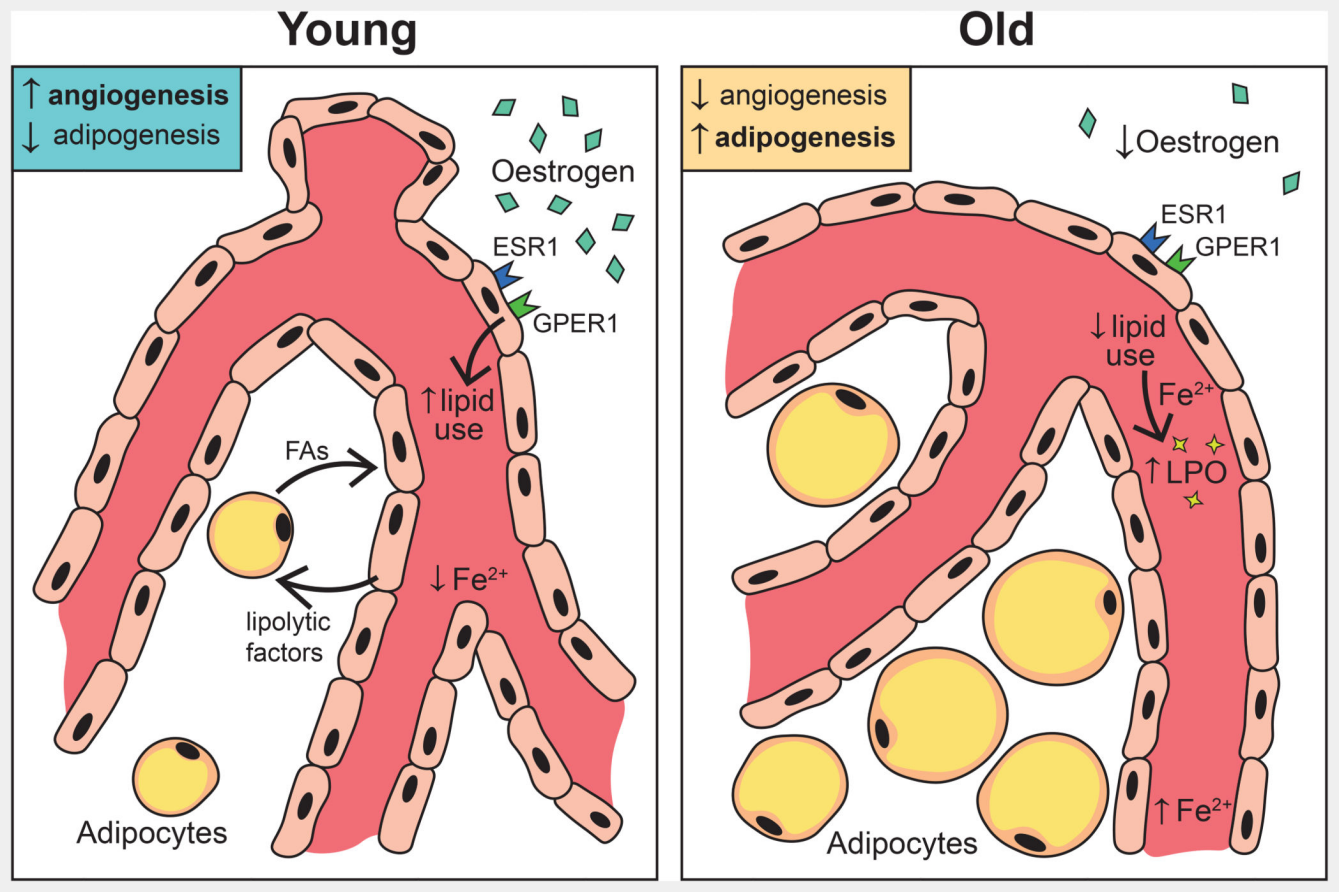
Schematic representation of key findings Oestrogen regulates the growth of blood vessels in bone through endothelial oestrogen receptor alpha (Esr1), and G-protein coupled oestrogen receptor-1 (Gper1). Oestrogen receptor signalling regulates endothelial cells’ use of fatty acid metabolism by promoting angiocrine release of lipolytic factors, enzymes and FA uptake from the microenvironment. Impaired lipid metabolism in aged endothelium and sex differences in endothelial Ferrous ion levels stimulate ageing phenotype in blood vessels of bones.

In this study, we find oestrogen plays an important role in mediating blood vessel growth by promoting fatty acid metabolism in BECs (Fig. 8). Genetic ER loss of function studies indicate constituent oestrogen signalling in bone vasculature of both male and female BECs and strongly argue for investigating their dose-dependency, interactions and redundancy in mediating downstream signalling.

Angiogenesis in actively growing bones is linked with limited adipocyte number, and the age-related decline in angiogenesis is associated with the accumulation of adipocytes in the bone. In women, oestrogen treatment has been identified to cause lipolysis and reduce bone marrow lipid content^43^. The role of oestrogen in regulating bone adipocytes has been widely recognised. In this study, we show a previously unknown association between blood vessels and adipocytes. We found that inhibition of lipid metabolism in BECs led to increased adipocytes in the bone microenvironment. In menopausal animal models, reduction of angiogenesis is associated with the accumulation of adipocytes, similar to ageing in humans.

Our data propose that oestrogen could regulate metabolic coupling to maintain energy balance in the bone microenvironment by driving lipolysis for the growth of blood vessels. Tissue-specific nature of vascular ageing^44^ is still in the initial stages of understanding. We find intrinsic ferrous ion levels could accelerate the ageing of BECs and contribute to bone loss observed in ageing females compared to males. The iron-dependent, non-heme nature of lipoxygenases corroborates the importance of iron in endothelial LPO levels. Overall, oestrogen is a key factor that limits LPO accumulation in BECs. Combined effects of reduced oestrogen, ferrous ions and LPO contribute to accelerated ageing in females. This study supports the fundamental notion of investigating sex differences in the vasculature to understand gender bias observed in the incidences of age-related cardiovascular diseases. Findings from this study identify LPO as a downstream target of oestrogen and suggest targeting LPO could be an alternative strategy to HRT to treat postmenopausal bone loss and bone repair. Moreover, the fundamental knowledge of bone endothelial sex differences can be beneficial in developing precision medicine against blood and bone diseases.

## Methods

### Mice

All animal experiments were carried out in accordance with the institutional guidelines and laws, following the protocols approved by the Imperial College London Animal Welfare and Ethical Review Body and the Home Office UK. All wildtype experiments were performed in C57BL/6J mice and were age- and sex-matched. All transgenic mice were age- and sex-matched for each experiment.

For the pregnancy experiments, the onset of pregnancy was determined by the presence of a vaginal plug. Mice were culled on relevant days post identification of vaginal plug along with virgin females and male mice as controls.

For hormonal treatments, mice were injected s.c. either with 2 μg 17β-oestradiol (Acros Organics), 1 mg Progesterone (Acros Organics) or 100 μg DHT (TCI) dissolved in ethanol and corn oil (100 μl). Vehicle mice received the equivalent dose of ethanol in corn oil. Pups (p10) were given 10 intermittent injections, and aged mice (65-75 wk) received 3 injections per week for 6 weeks. Mice injected with 17β-oestradiol were injected intraperitoneally with (250mg/ml) 5-ethynyl-2′-deoxyuridine (EdU) 3h prior to culling to identify endothelial cells undergoing proliferation. Pups (P10) received 10 doses of 10 μg of Letrozole (Sigma-Aldrich) in DMSO for anti-aromatase treatment.

For the murine menopause model, female mice were injected with 160mg kg^-1^ day^-1^ with 4-vinyl cyclohexene diepoxide (Fluorochem) for 20 days and left for a further 32 days to induce complete follicular atresia. Vaginal samples were taken daily to confirm persistent dioestrus. Control mice were injected with PBS.

Ovariectomised (OVX) mice were purchased from Charles River Laboratories, UK. Ovariectomy was performed on C57BL/6J wildtype mice. E2 cohorts received 17β-oestradiol two weeks after the surgery for 4 weeks (3 injections/week), while control cohorts received vehicle injections.

Deferoxamine mesylate (DFM) was administered at 25 μg/g weight of mice i.p. for two weeks every alternate day. Mice were sacrificed the day after the last injection for analysis. To inhibit Cpt1a, Etomoxir at 30mg/kg was injected intraperitoneally for 10 days and mice were sacrificed on P28.

For endothelial-specific deletion of oestrogen receptors, conditional *Esr1^lox/lox^, Esr2^lox/lox^ or Gper1^lox/lox^* mice were bred with *Cdh5(PAC)-CreERT2* transgenic mice. Cre activity was induced by Tamoxifen (80mg/kg) injections from P10-P14, and mice were analysed at P28. Littermate Cre-mice were also injected with Tamoxifen and used as controls. Similarly, conditional *Cpt1a^lox/lox^* mice were bred with *Cdh5(PAC)-CreERT2* transgenic mice for endothelial-specific deletion and mice were analysed at P28. To induce Cre activity at the adult stage, mice were injected from P66-P70 and culled on P84.

To induce the generation of lipid peroxides in young mice, pups (P10) were given 5 intermittent i.p. injections of Artemisinin (TCI, 200mg/kg) in DMSO. Aged mice received 15 doses of Liproxstatin-1 (Tocris Bioscience, 10mg/kg) i.p. for 4 weeks to inhibit lipid peroxide production.

### Bone Processing, Immunohistochemistry & Confocal Microscopy

Tibiae were dissected and cleaned and underwent immediate fixation for 4 hrs on ice in 4% paraformaldehyde solution (PFA), followed by decalcification with 0.5M EDTA with constant agitation at 4°C. Bones were then kept in cryopreservative solution (20% (w/v) sucrose, 2% (w/v) polyvinyl pyrrolidine (PVP)) solution for a further 48h. For cryo-sectioning, bones were embedded in 8% (w/v) gelatin, 20% (w/v) sucrose, 2% (w/v) PVP. A Leica cryostat was used to generate 70 μm sections on microscopic slides used for immunostaining.

For phenotypic analyses of tibiae by immunohistochemistry, bone sections were hydrated with PBS, followed by permeabilisation with 0.25% Triton-X for 10 mins and blocking with 5% donkey serum (DS) for 30 mins. Primary antibody was diluted in 5% DS and incubated for 2.5 hr at RT or overnight at 4°C. Primary antibodies used: Endomucin (Santa Cruz Biotechnology; 1:100), Osterix (Abcam; 1:600), CD31 (R&D Systems; 1:100), Perilipin (Cell Signalling Technology; 1:100), Perilipin (GeneTex, 1:100), anti-Ki67 (Abcam, 1:100), Laminin (Sigma; 1:100), Fabp4 (R&D Systems, 1:100), anti-4-Hydroxynonenal (Abcam, 1:200), HSL (Cell Signalling Technology; 1:50), ATGL (Cell Signalling Technology; 1:100), CD45 (BD Pharmingen, 1:100), Itgb3 (Cell Signalling Technology; 1:100).

Slides were then washed three times with PBS for 5 mins each and incubated with fluorophore-conjugated secondary antibody diluted in 5% DS for 1 hr at RT. Secondary antibodies used: Anti-rat Alexa Fluor 594 (Invitrogen; A21209, 1:400), anti-rat Alexa Fluor 488 (Invitrogen; A21208; 1:400), anti-goat Alexa Fluor 488 (Invitrogen; A11055, 1:400), anti-goat Alexa Fluor 546 (Invitrogen; A11056; 1:400), anti-goat Alexa Fluor 647 (Invitrogen; A21447; 1:400), anti-rabbit Alexa Fluor 488 (Invitrogen; A21206; 1:400), anti-rabbit Alexa Fluor 546 (Invitrogen; A10040; 1:400) and DAPI for nuclear staining. Slides were washed three times with PBS for 10 mins each and then mounted with a coverslip using FluoroMount G solution (Southern Biotech).

EdU labelling was performed by Click-iT chemistry (Invitrogen, C10340) following manufacturer’s guidelines, following primary and secondary antibody incubations.

High-resolution 3D images were acquired using a Leica TCS SP8 confocal laser scanning microscope using Leica LASX software.

### Micro-Computed Tomography analysis

We collected fresh tibiae and femur from mutants and their littermate controls or from control and treated mice for phenotype analysis using microcomputed tomography (micro-CT). Freshly isolated bones were cleaned thoroughly and fixed in 4% paraformaldehyde. For analysing mineralised regions in bones, fixed bones were used for microCT scanning. For lipids analysis, we performed osmium tetroxide labelling. Briefly, fixed bones were decalcified in EDTA solution for 3-10 days and stained with 2% osmium tetroxide solution for 48 hours. Bones were then washed thoroughly and stored till analysis. The bones were scanned using cabinet cone-beam micro-CT (μCT 100, Scanco Medical AG, Switzerland) and software IPL V5.15 at Scanco Medical AG, Switzerland. A voxel size of 10 μm was chosen in all three spatial dimensions. For each sample, at least 400 slices were evaluated, covering more than 4mm at a voltage of 70 kVp, intensity 114 μA, 8W and integration time 800 ms. For evaluation, 3mm length below the growth plate is chosen as the volume of interest.

### Isolation & culture of primary murine Bone Endothelial Cells (BECs)

Tibiae and femurs were dissected and collected in ice-cold PBS and washed subsequently with sterile PBS under sterile conditions. Bones were crushed with a mortar and pestle, and the remaining bone debris was digested with collagenase for 20 mins at 37°C and filtered together to obtain a single cell suspension. RBC lysis (ChemCruz) was performed (5 mins, RT) to eliminate RBCs, and total BM cells were pelleted and kept on ice.

BECs were isolated using Dynabeads® sheep anti-rat IgG (Invitrogen) following the manufacturer's protocol. Briefly, beads were first coated with Rat Endomucin (Santa Cruz Biotechnology) for 45 mins on a rotator at 4°C, washed with isolation buffer (0.1% FBS-PBS) on a magnetic rack and then incubated with total cells for 30 mins on a rotator at 4°C to isolate endothelial cells only. The mixture was washed with isolation buffer on a magnetic rack to isolate a positive selection of Endomucin expressing cells and plated in fibronectin-coated (Sigma-Aldrich) cell culture plates in EBM-2 basal medium (Lonza) supplemented with EGM-2 growth medium (2% FBS, 0.4% hFGF-B, 0.1% VEGF, 0.1% R3-IGF-1, 0.1% Ascorbic Acid, 0.1% hEGF, 0.1% Heparin, 0.04% Hydrocortisone). Cells were passaged every 3-4 days using 0.25% Trypsin-EDTA (Sigma-Aldrich).

### *In vitro* assays

#### Nutrient supplementation

BECs grown in EGM2 complete medium for 1 day were used for this experiment. After washing with HBSS, cells were treated either with vehicle (DMSO) or 17β-Oestradiol (10μM) for 16 hrs in Glucose-, Glutamine- and serum-free endothelial cell medium (cell biologics) and supplemented either with 5mM Glucose, 2mM Glutamine or 20mM Linoleic acid-Oleic acid (Sigma) to determine the effect of nutrient supplementation on cell growth. Cells were stained with anti-Ki-67 (Abcam, 1:300) to measure EC proliferation or with cleaved caspase-3 (Cell Signalling Technology, 1:300) to measure apoptosis.

#### Lipid uptake

BECs cultured on coverslips were treated with vehicle (DMSO) or 17β-Oestradiol for 60 hrs and then either incubated with Bodipy 558/568 C12 (ThermoFisher, 1 μM) or stained with HCS LipidTOX Green (ThermoFisher, 1:1000) for 30 min at 37°C. Cells were washed and mounted on slides and imaged using a Leica TCS SP8 confocal microscope.

#### Cpt1a inhibition

BECs cultured on coverslips were treated with vehicle (DMSO) or 17β-Oestradiol along with etomoxir (100μM). Cells were stained with anti-Ki-67 (Abcam, 1:300) to measure EC proliferation or with cleaved caspase-3 (Cell Signalling Technology, 1:300) to measure apoptosis.

#### Fabp4 inhibition

BECs cultured on coverslips were treated with vehicle (DMSO) or 17β-Oestradiol along with anti-fabp4 BMS-309403 (10μM). Cells were stained with BODIPY 558/568 C12 to determine whether lipid uptake was reduced.

#### Gper1 signalling inhibition

BECs cultured on coverslips were treated with vehicle (DMSO) or 17β-Oestradiol along with G36 (Tocris Bioscience, 10 μM) for 60 hrs and then stained for Bodipy 558/568 C12 as explained for lipid uptake.

#### Iron assay

BECs were cultured from male and female bones separately and treated with vehicle (DMSO) or 17β-Oestradiol for 60 hrs before the assay. BECs were collected by trypsinisation and centrifugation and lysed by sonication, and resuspended in iron assay buffer (Abcam, ab8366.) The assay was performed as per manufacturer guidelines for the Iron (II) assay with OD measured at 593 nm and Iron (II) content determined by a standard curve.

#### LPO assay

Lipid Hydroperoxides generated by ferrous ion redox reactions were measured by a colourimetric assay (Cayman Chemical, 705003) following the manufacturer's protocol. In brief, total ECs were isolated from the bones of young and aged mice treated with vehicle or E2 as explained previously using Dynabeads and lysed by sonication upon bead removal. A methanol: chloroform extraction followed, taking the chloroform layer for reaction with thiocyanate ions to detect lipid hydroperoxides generated by measuring OD at 500 nm using FLUOstar Omega Microplate reader 3.0.

#### 17β-oestradiol ELISA

The concentration of 17β-Oestradiol was determined from blood serum by an ELISA (Abcam, ab108667) according to the manufacturer's protocol. Circulating blood was collected from mice and allowed to clot for 45-60 mins at RT and centrifuged at 3500 rpm for 10 mins to obtain serum. The l7β-Oestradiol-HRP conjugate was added to samples or standards and incubated for 2 hours at 37°C. Following washing of all samples, TMB substrate was added and incubated for a further 30 mins at RT. The reaction was halted by the addition of a stop solution, and OD was measured at 450nm to quantify E2 concentration using a standard curve.

#### ELISA

Supernatant was collected from cultured BECs treated with vehicle (DMSO) or l7β-Oestradiol to measure the concentration of TNFα (R&D Systems, MTA00B) and IL-6 (R&D Systems, M6000B) released using ELISA according to manufacturer's protocol. Specifically, assay diluent was added to standards or samples and incubated for 2 hours at RT. Following washing of all samples, respective protein conjugate was added for 2 hours, following further washing and incubation with substrate solution for 30 mins. OD was measured at 540nm and 450nm (subtracted for wavelength correction), and Protein concentrations were determined by a standard curve.

### Flow Cytometry & Analysis

Femurs were dissected out and crushed with a mortar and pestle in ice-cold PBS. Bone marrow solution was passed through a 100μm filter and pelleted. RBC lysis (ChemCruz) followed as explained above. Cells were resuspended in PBS and stained for total endothelial cells: primary antibody CD45 (BD Pharmingen; 1:100) or type H ECs: primary antibody Endomucin (Santa Cruz Biotechnology, 1:50) and secondary antibody Brilliant Violet-421 (Jackson ImmunoResearch; 1:50) along with CD31-PE (Miltenyi Biotech; 1:20) or CD31-APC (Miltenyi Biotech; 1:20). Mitochondrial staining was performed following antibody incubations with MitoSOX Thermo Fisher Scientific, 1 μM for 15 mins at 37°C. Cells were run through the BD LSRII flow cytometer using software BD FACSDiva 9.0.0.

For Fabp4 staining, Fabp4-AF488 (Santa Cruz Biotechnology, 1:50) was added along with CD31-PE following primary CD45 incubation.

For Ki-67 staining, cells were stained as explained above for total endothelium. Following secondary antibody staining and washes, cells were fixed, permeabilised (0.1% BSA, 0.01% Saponin) and stained with FITC anti-mouse Ki-67 (Biolegend; 1:75) for 45 mins RT, and cells were acquired on the BD LSRII flow cytometer.

To detect intracellular ferrous ions, cells were incubated with FeRhoNox-1 (Goryo chemical, 5 μM) for 15 mins at 37°C following staining for total endothelial cells.

BECs in culture, treated with Vehicle (DMSO) or 17β-Oestradiol (10μM) were trypsinised, pelleted and incubated with either BODIPY 558/568 C12 (ThermoFisher, 1 μM) or HCS LipidTOX Green (Thermofisher, 1:1000) for 30 min at 37°C prior to acquisition.

For the lipid peroxide sensor, cells were stained for total endothelium and then incubated with BODIPY 665/676 (ThermoFisher, 2 μg/ml) for 30 min at 37°C before acquisition.

All flow cytometry analysis was done using FlowJo 10.0.0 Software. Total endothelial cells were gated for CD31+ CD45-Ter119-populations, and type H ECs were gated for Endomucin^high^ CD31^high^ populations.

### Metabolic Assays

#### Radioactive Assays

BECs were seeded at approximately 25000 cells/well for all radioisotope labelling experiments and were treated with Vehicle (DMSO) or 17β-Oestradiol for 48h prior to radiolabelling. To measure glucose and glutamine oxidation, cells were incubated with 0.3 μCi/ml D-[6-^14^C]-Glucose (Perkin Elmer) or 1.1 μCi/ml L-[^14^C(U)]-Glutamine (Perkin Elmer) in cell culture medium for 6h. To stop cellular metabolism, 250μl of Perchloric acid was added to each well with the addition of a filter paper soaked in hyamine hydroxide to absorb ^14^CO_2_ released by glucose or glutamine oxidation overnight at room temperature. Radioactivity was determined by liquid scintillation counting to denote disintegrations per minute. For the fatty acid oxidation study, etomoxir was used to inhibit Cpt1a as described before. To measure palmitate oxidation, cells were incubated with 2 μCi/ml [9,10-^3^H]-Palmitic acid (Perkin Elmer) in the cell culture medium for 6h. The cell culture medium was transferred to glass vials with hanging filter paper to absorb ^3^H_2_O released over 48h at 37°C. Radioactivity was determined by liquid scintillation counting (QuantaSmart) to denote disintegrations per minute.

### Image processing and quantification

Z-stacks of images were reconstructed in 3D and analysed using Imaris Image Analysis Software version 9.0.0. Perilipin+ and Type H area quantification was done using ImageJ (Fiji) on the maximum projection of Z-stacks. Osx+ cell numbers were automatically counted using the spots detection feature by setting a threshold diameter for Osx+ nuclei to exclude background using Imaris. Type H vessel volume was quantified using the surfaces feature to generate 3D overlaps of Emcn+ CD31+ volumes in Imaris. EdU+ endothelial cells were automatically counted using the spots feature detection in Imaris, similar to Osx+ nuclei, after applying a mask for Emcn+ blood vessels using the surfaces feature. Ki67+ endothelial cells were quantified similarly. Mean fluorescence intensity (MFI) for Emcn+ 4HNE and Fabp4 was automatically generated during the 3D reconstruction of z-stacks for volume analysis by creating a mask for Emcn+ vessels. CD31+ Ki-67+ cell nuclei were counted using the cell counter function in ImageJ (Fiji). CD31+ 4HNE (MFI) for cells were generated in ImageJ (Fiji) by using the integrated density and mean grey values. Background fluorescence was subtracted to obtain corrected fluorescence intensity per cell across several images. Plin+ ATGL and HSL (MFI) were calculated in a similar way using integrated density and mean grey values per lipid droplet across several images for 4-5 bone samples.

### qRT-PCR

ECs were isolated with antibody-coated beads, as explained above. Instead of plating cells for culture, cells were lysed with RLT buffer and RNA was extracted using the RNeasy Plus Micro Kit (Qiagen, 74034) as per manufacturer's guidelines. Total RNA was converted to cDNA using the iScript cDNA Synthesis Kit (Bio-Rad) according to kit instructions. Diluted cDNA was used for qPCR performed using SYBR Green Master Mix (ThermoFisher Scientific) with primers for the following genes: *Tgfb*, *Eng*, *Tie1*, *Pecam1*, *Itga5*, *Flt4*, *Flt1*, *Fgfr2*, *Sox18*, *Nrp1*, *Adipoq*, *Fabp4*, *Cyp1b1*, *Lepr*, *Aldh1a2*, *TNFα*, *Il6*. β-*actin* was used as a housekeeping gene.

To confirm the loss of gene function at the transcript level for gene deletion experiments, ECs were isolated from femurs of tamoxifen-injected transgenic mice for qPCR using primers for each gene (*Esr1*, *Esr21*, *Gper1*, *Cpt1a*). Primer sequences used in this study are provided in a supplementary table 1.

Reactions were run on a CFX real-time PCR machine (Bio-Rad) using CFX manager 3.1 software. Data were calculated using the ΔΔCt method to show fold gene expression or relative mRNA levels.

### RNA sequencing

Isolated bone ECs were lysed using RLT buffer (Qiagen). RNA was extracted using the RNeasy Plus Micro Kit (Qiagen, 74034) as per the manufacturer's guidelines.

#### Library preparation

The quality of RNA for each individual sample was checked using a 2100 Bioanalyser (Agilent). The top three samples from each set based on RNA quality were processed further for library preparations. TruSeq Total RNA Library Preparation Kit (Illumina) was used to prepare libraries following the manufacturer's guidelines. Libraries were sequenced at LMS Genomics Facility on an Illumina HiSeq 2500 platform with 100-nucleotide-length paired-end reads.

#### Data Analysis

Paired-end 100bp RNAseq reads aligned using the mouse genome assembly GRCm39 STAR (version=2.7.10a) ^45^. Gene-based read count was obtained using the featureCounts version (Version 2.0.1) ^46^. The count data were normalised using the Variance Stabilizing Transformation (VST) function from the DESeq2 package^47^ for visualisation by PCA. Differential gene expression analysis between the male versus females for each age group was performed using DESeq2. Differentially expressed genes were selected (protein coding) using an FDR-adjusted *P* value<0.05. Further, the Ensembl IDs were annotated to gene symbols and Entrez Gene ID using biomaRt^48^. The differentially regulated genes list is provided in the supplementary Dataset 1. Gene-set enrichment analysis was performed using Generally Applicable Gene-set Enrichment (GAGE) (version 2.42.0) ^19^. For functional annotation, we used gene sets from KEGG database. The gage analysis was performed on differentially regulated genes from 65 weeks of data, where the female sample was taken as a reference sample. The Significant KEGG terms were selected using a *P-value* < 0.05.

### Statistical Methods

GraphPad Prism v9.4.1 was used for generating all graphs and for statistical analysis. Statistics performed for each figure are mentioned in the corresponding figure legend. Where indicated student’s two-tailed unpaired t-tests, one-way- or two-way analysis of variance (ANOVA) following Tukey’s multiple comparison test were performed for data sets. Error bars for biological data indicate mean ± s.d. and for numerical quantification indicate mean ± s.e.m. *P* values <0.05 was considered statistically significant. Sample sizes were selected based on previous experiments and published data. All data were generated using at least two independent experiments to reproduce similar results.

## Extended Data

## Supplementary Material

Rodrigues_SourceData_ED_Fig1

Rodrigues_SourceData_ED_Fig2

Rodrigues_SourceData_ED_Fig3

Rodrigues_SourceData_ED_Fig4

Rodrigues_SourceData_ED_Fig5

Rodrigues_SourceData_ED_Fig6

Rodrigues_SourceData_ED_Fig7

Rodrigues_SourceData_ED_Fig8

Rodrigues_SourceData_ED_Fig9

Rodrigues_SourceData_ED_Fig10

Rodrigues_SourceData_Fig1

Rodrigues_SourceData_Fig2

Rodrigues_SourceData_Fig3

Rodrigues_SourceData_Fig4

Rodrigues_SourceData_Fig5

Rodrigues_SourceData_Fig6

Rodrigues_SourceData_Fig7

Supplementary Data Set 1

Supplementary information

Supplementary Table 1

## Data Availability

RNA-seq data generated for this study have been deposited in the National Center for Biotechnology Information Gene Expression Omnibus (GEO) database under accession numbers ***GSE163515*** and ***GSE180246***. Additional data supporting the findings in this study are included in the main article and associated files. Source data are provided with this paper.
