# Supplementary material for: Oestrogen enforces the integrity of blood vessels in the bone during pregnancy and menopause
Source: Nat Cardiovasc Res. Author manuscript; Available in PMC 2022 Dec 16. (PMC7613952; doi:10.1038/s44161-022-00139-0)
Supplement: Supplementary information [file EMS153774-supplement-Supplementary_information.pdf]

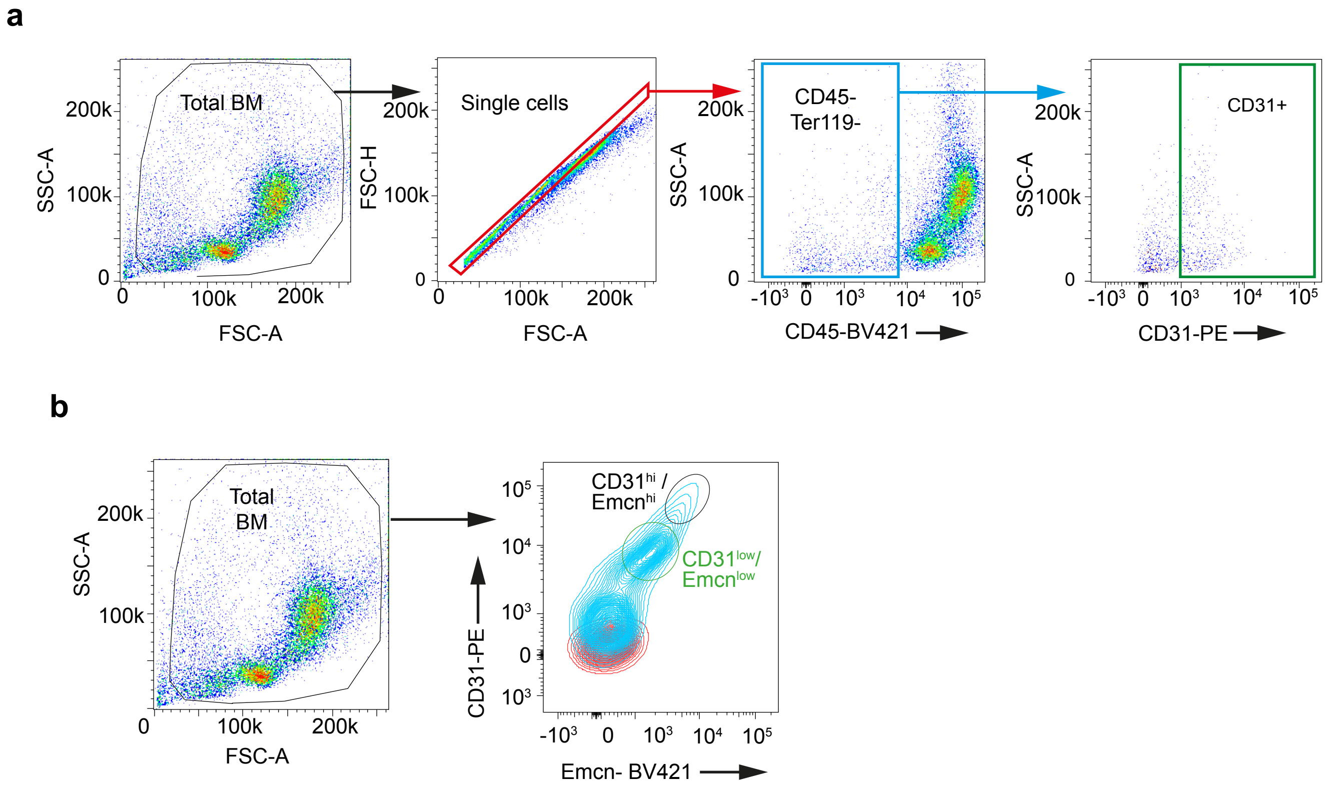

Supplementary Figure 1: Flow cytometry gating strategy

a, Flow cytometry plots showing gating strategy for total ECs (CD31+ CD45- Ter119-).

b, Flow cytometry plots showing gating strategy for type-H ECs (CD31high Emcnhigh).

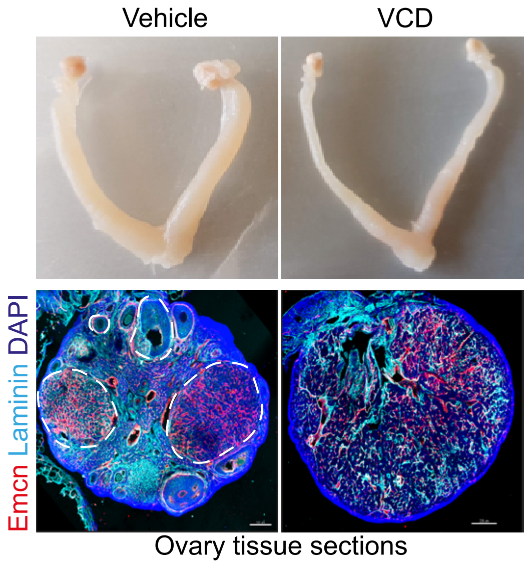

Supplementary Figure 2: Depletion of ovarian follicles by VCD treatment
Representative dissected ovaries attached to uterine horns of Vehicle and VCD-administered female mice, illustrating smaller VCD ovaries with flaccid uterine horns. Confocal images of ovary tissue sections from Vehicle and VCD mice (n=5 mice) show depleted ovarian follicles in VCD ovaries. Scale bars 200um
